# Allometry of cell types in planarians by single-cell transcriptomics

**DOI:** 10.1126/sciadv.adm7042

**Published:** 2025-05-07

**Authors:** Elena Emili, Alberto Pérez-Posada, Virginia Vanni, David Salamanca-Díaz, Dianalí Ródriguez-Fernández, Maria D. Christodoulou, Jordi Solana

**Affiliations:** ^1^ Department of Biological and Medical Sciences, Oxford Brookes University, Oxford, UK.; ^2^ Living Systems Institute, University of Exeter, Exeter, UK.; ^3^ Department of Biosciences, University of Exeter, Exeter, UK.; ^4^ Department of Statistics, University of Oxford, Oxford, UK.

## Abstract

Allometry explores the relationship between an organism’s body size and its various components, offering insights into ecology, physiology, metabolism, and disease. The cell is the basic unit of biological systems, and yet the study of cell-type allometry remains relatively unexplored. Single-cell RNA sequencing (scRNA-seq) provides a promising tool for investigating cell-type allometry. Planarians, capable of growing and degrowing following allometric scaling rules, serve as an excellent model for these studies. We used scRNA-seq to examine cell-type allometry in asexual planarians of different sizes, revealing that they consist of the same basic cell types but in varying proportions. Notably, the gut basal cells are the most responsive to changes in size, suggesting a role in energy storage. We capture the regulated gene modules of distinct cell types in response to body size. This research sheds light on the molecular and cellular aspects of cell-type allometry in planarians and underscores the utility of scRNA-seq in these investigations.

## Teaser

Single-cell RNA sequencing allows the exploration of how cell types vary in proportion with body size in asexual planarians.

## INTRODUCTION

Allometry studies how body parts, structures, or processes in an organism relate to its overall body size (*1*). It is important for understanding ecology, physiology, metabolism, disease, and other aspects of organismal biology. Scaling laws often govern allometry, such as the Kleiber law that relates metabolism to body size using a three-fourth power function (*2*).

Given that tissues, organs, and body parts scale allometrically, cell types—the building blocks—must also scale allometrically. Cells can be classified in different types based on their morphology, function, or gene expression patterns (*3*). However, the exact definition of a cell type is still a topic of debate and controversy among researchers (*4*–*10*). Despite the importance of cell types in understanding biological systems, the study of how cell types vary with body size is still a largely unexplored field. We refer to this study as cell-type allometry, encompassing changes in cell-type proportions, morphology, and gene expression, as well as the existence of size-dependent cell types. Advances have been made using morphological cell-type classifications and markers (*11*), but these are typically limited to one or few cell types. Whether cell types vary with body size, and how, is still a question, for most cell types, in most organisms.

The study of cell types has been enhanced by single-cell methods (*12*–*14*), particularly single-cell RNA sequencing (scRNA-seq) (*15*–*17*), which measures the expression of hundreds to thousands of individual transcripts in thousands of cells. Each cell type is characterized by specific expression of effector and regulatory genes, allowing clustering algorithms to reveal the presence of cell populations. However, it is still unclear how these populations correspond to cell types as opposed to other cell states such as stress, differentiation, or developmental states. Despite this, scRNA-seq enables quick and simple classification of cells into largely interpretable populations, such as neurons, muscle cells, and epidermal cells.

Single-cell analysis techniques have potential for studying cell-type allometry, but several challenges remain to be addressed. Cell dissociation techniques can cause stress responses and biases (*18*–*20*), leading to cell death and differential survival. Including different samples can also lead to batch effects. However, fixative cell dissociation approaches such as acetic-methanol (ACME) (*21*) can address the first concern, while combinatorial single-cell transcriptomic approaches such as Split pool ligation-based single-cell transcriptome sequencing (SPLiT-seq) (*21*, *22*) allow for sample multiplexing and convenient multisample experiments. Combining ACME and SPLiT-seq could therefore enhance the study of cell-type allometry.

Planarians are an interesting model for studying cell-type allometry because they exhibit a wide range of body sizes, with sexual populations typically being larger than asexual ones. Further to that, planarians grow when they are fed and can degrow when starved (*23*–*25*). These processes follow allometric scaling rules (*11*, *26*–*32*). Romero and Baguñà examined cell dissociations from animals of varying sizes to study 13 basic cell types based on their morphology (*26*). They showed that body size increases mostly by increases in cell numbers, with quantitative changes in the proportions of cell types. For example, larger planarians have a decrease in neuron proportion and an increase in fixed parenchymal cells. Thommen and coworkers (*27*) established that the metabolic rate in planarians also follows the Kleiber law. Larger planarians have more cells, but their cellular size scales with mass following a three-fourth power relationship. This indicates that larger planarians have many more cells, but at least some of these cells are also larger. It remains unknown if this applies to all cell types or if it is largely driven by one or few cell types. Beyond previous allometric studies, planarians are an excellent model for developmental biology and regeneration, and plenty of cell types have been described at the cellular or molecular level, including several types of neurons (*33*), muscle (*34*, *35*), and the different cell types that make up planarian organs such as the protonephridia (*36*, *37*), the pharynx (*38*), and the planarian gut (*39*–*41*), including a recently found cell type, the basal cells (*39*). Planarians are also an excellent model for single-cell biology (*21*, *42*–*49*), which has fueled cell-type research in planarians. However, the allometry of their cell types has not been investigated yet with these methods.

Here, we aimed to investigate the allometry of cell types in asexual planarians using scRNA-seq across body size categories. To minimize batch effects and enable an integrated analysis, we used SPLiT-seq to multiplex planarians of different sizes in a single experiment. Our findings reveal that asexual planarians of different sizes are made of the same cell types in different proportions. Specifically, smaller planarians exhibit a higher proportion of neurons and fewer parenchymal cells. Unlike previous studies, scRNA-seq has greater resolution on which types of neurons and parenchymal cells vary the most. Notably, further to cell proportions, scRNA-seq allows us to access cell type–specific differential gene expression. Our data show that epidermal cells and the recently found basal cells are the ones that respond more dynamically to organism size at the transcriptomic level. Overall, our results show that scRNA-seq using ACME and SPLiT-seq is a powerful method to study cell-type allometry that can be applied to virtually any organism. Our data reveal the basic principles of cell-type scaling at the cellular and molecular level.

## RESULTS

### A single-cell approach allows studying cell-type allometry in planarians of different body sizes

**Fig. 1.**
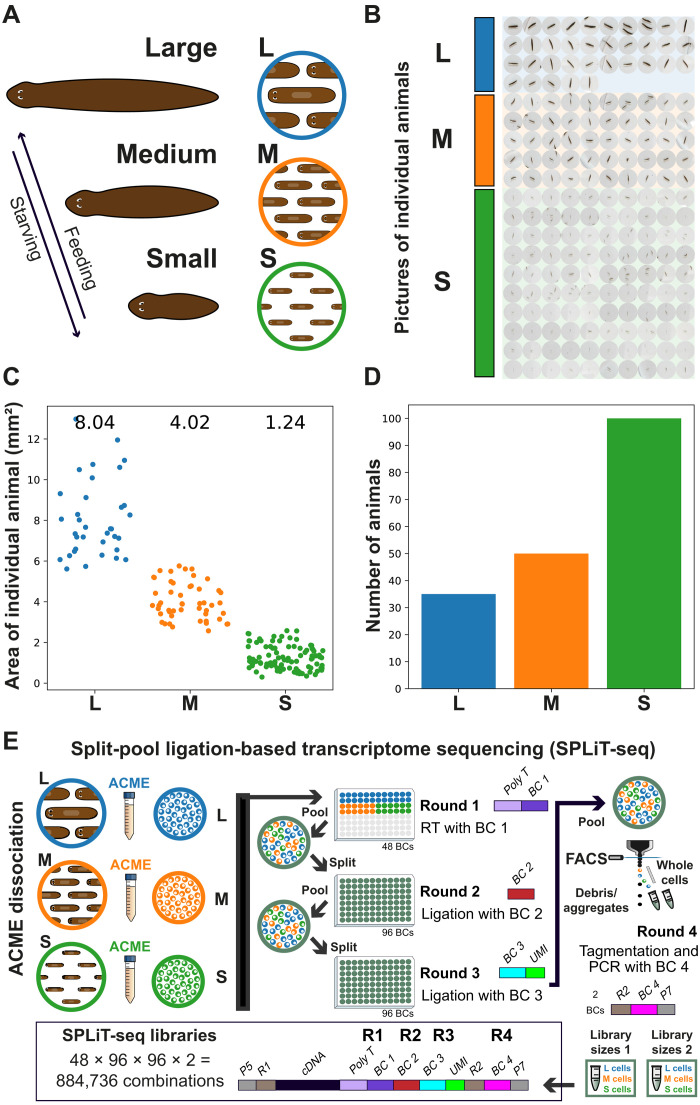
Animal selection, measurement, and single-cell transcriptomic approach. (**A**) Cartoon depicting planarian growth and degrowth, following allometric rules. (**B**) Low-resolution images of selected animals taken with a standard field camera. (**C**) Area of individual animal per size class. (**D**) Number of animals per size class. (**E**) Experimental workflow. S, M, and L planarians were ACME dissociated in separate tubes and subjected to multiplexed combinatorial barcoding for sequencing using SPLiT-seq.

We aimed at generating one scRNA-seq experiment to multiplex asexual planarians of different sizes (Fig. 1A). We first selected asexual planarians from our cultures and classified them in sizes by visual inspection. Planarians not only grow when they are fed but also degrow when they are starved. In our mixed cultures both processes might be happening simultaneously. To decouple size from feeding and starvation conditions, we selected planarians at random and classified them only by size. To corroborate our inspection, we took low-resolution images using a general field camera and measured the area of each individual using Fiji (Fig. 1B) (*50*). This was done to minimize the stress of monitoring the animals under a bright light before single-cell transcriptomics. We undertook the image capture immediately before cell dissociation to avoid that animals change in size further after capture and to prevent fission events that would modify the sizes. We used ACME (*21*) to dissociate the different sized animals in four independent cell dissociations. We initially aimed at classifying four body sizes but found that the two larger size classes overlapped substantially and decided to consider them as one class. We therefore considered three classes of body size, termed large (L), medium (M), and small (S). Measuring the area of the individual animals revealed a good size separation in three classes (Fig. 1C and data S1). The mean size of our classes was 8.04, 4.02, and 1.24 mm^2^ for L, M, and S planarians, respectively. The largest planarian measured 12.98 mm^2^, and the smallest measured 0.30 mm^2^, encompassing a ~ 43-fold difference in area. In the average populations, the fold difference between L and S planarians was 6.45. To achieve comparable cell numbers, we used 35, 50, and 100 animals of L, M, and S sizes, respectively (Fig. 1D).

We then subjected these cell dissociations to scRNA-seq using SPLiT-seq (*21*, *22*). Essentially, SPLiT-seq barcodes cells in different split/pool rounds, so that single cells are labeled with combinations of barcodes that are unique. The first barcoding round can be used to multiplex different samples (Fig. 1E). This step allows demultiplexing the cells coming from each sample in the data analysis steps. After this first round, cells are pooled, and every subsequent barcoding, sorting, and library preparation step is performed in pools of cells containing all samples, therefore minimizing batch effects. Furthermore, SPLiT-seq is very robust against ambient RNA, as the successive pooling and centrifugation steps select the material encapsulated in cells, while the supernatants (with any soluble ambient RNA released from the cell suspension) are eliminated in each round. We implemented fluorescence-activated cell sorting (FACS) after the third round of combinatorial barcoding, a step that allows us to sort barcoded whole cells into the lysis buffer, before the fourth round of barcoding. This step notably reduces the presence of aggregates, broken cells, and particles of cellular debris in the experiment. We obtained two sublibraries that were subjected to the fourth round of barcoding during library preparation. Under these combinatorial conditions, a total of 884,736 barcode combinations are theoretically possible. The cost of a single SPLiT-seq experiment is around ~£750 in enzymes, FACS sorting time, and library preparation reagents, making it an affordable scRNA-seq approach. We sequenced the library pool using Illumina NovaSeq technology to obtain 715 million paired-end reads.

### A cell-type atlas of planarian body sizes reveals cell-type proportions at single-cell resolution

**Fig. 2.**
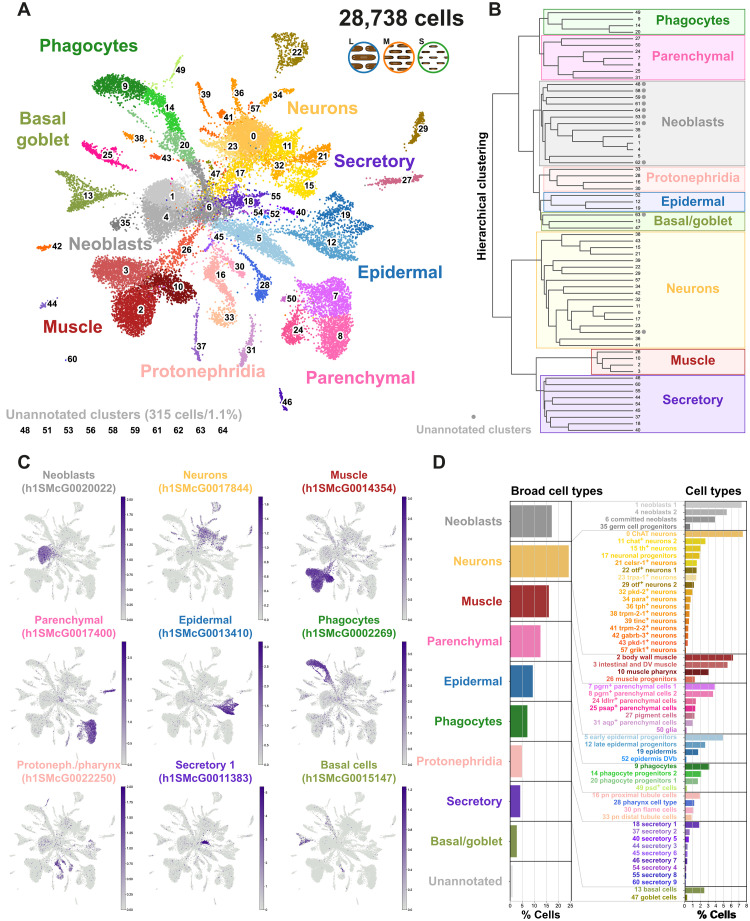
The *S. mediterranea* size dataset. (**A**) UMAP visualization of the *S. mediterranea* size single-cell atlas with clusters colored according to their cell cluster classification. (**B**) Hierarchical clustering dendrogram of cell clusters to define broad group identities. Gray dots denote the unannotated clusters. (**C**) UMAP features plots of markers of the major broad types. (**D**) Cell cluster percentage over the total at the broad cell groups and the cell cluster levels.

To analyze SPLiT-seq libraries, we processed the reads obtained through our SPLiT-seq analysis pipeline (*21*, *51*) using a new version of the *Schmidtea mediterranea* genome (*52*). Similar to previous SPLiT-seq experiments, this resulted in relatively shallow single cell transcriptomes in terms of unique molecular identifiers (UMIs, mean 297 UMI per cell). SPLiT-seq, however, is more robust to ambient RNA compared to droplet methods, and this results in good clustering resolution even with relatively shallow UMI contents. The final dataset contained 28,738 cells, consistent with the ~19Kx2 cells observed in each of the two FACS-sorted samples. In these conditions, we expected ~3% of the cells being collisions, i.e., two cells that share the same barcode. Leiden clustering revealed the presence of ~40 to 73 cell clusters, depending on clustering resolution. We selected resolution 3, which resolved 64 cell populations, largely corresponding to planarian well-known cell types. We represented those populations using uniform manifold approximation and projection (UMAP) (Fig. 2A) and grouped these cell-type identities in broad groups by hierarchical clustering (Fig. 2B). We annotated and named these cell clusters using single-cell atlas studies (Fig. 2C, data S2 and S3, and fig. S1) (*21*, *42*, *44*) and cross-referencing published markers with markers obtained from this experiment (data S4 to S6). Our dataset resolves three types of differentiated muscle cells, three epidermal and gastrodermal differentiation states (fig. S1), and up to 17 neuronal cell populations. This resolution reveals clusters containing relatively rare cell types such as the glia (0.19%; Fig. 2D and fig. S1), the *psd*^+^ cells (0.26%; Fig. 2D) but fails to resolve the photoreceptors that are clustered together with other cholinergic neurons. We annotated cluster 13 as the basal cells (Fig. 2, A to D), which had been clustered together with phagocytes and goblet cells in previous studies (*21*, *42*, *44*). Neoblasts were clustered in three major clusters, including one cluster that we called “committed neoblasts” as it expressed markers of differentiation to gastrodermal and epidermal types (fig. S1). Last, a number of unabundant cell types (1.1%; 10 clusters, 315 cells; Fig. 2, A and B, and data S4 to S6) had unspecific markers or no significant markers were interpreted as putative doublets and were left unannotated in the final dataset. The average mean counts and genes per cluster vary from cluster to cluster (fig. S2, A to D). To elucidate whether this variation is technical or reflects biological properties of the cells instead, we studied the relationship between counts and cells at the cell and cluster level. The number of genes detected in each cell largely correlates with the number of counts obtained, as expected (fig. S2E). However, at the cluster level averages, some clusters deviate from the correlation: Secretory cell types have more UMI counts than other cell clusters with similar gene counts, consistent with the idea that they express a few genes at higher levels (fig. S2F). The cluster with more gene counts and UMI counts is *nanos*^+^ germ cell progenitors, consistent with previous publications (*21*). These observations indicate that the variations in cluster UMI counts and genes are biological signals. Together, these results show that our ACME + SPLiT-seq approach reliably detects dozens of cell clusters.

### Different cell states are enriched in planarians of different body size

We aimed to determine if all body sizes have the same cell types, building on previous studies that found no evidence of size-dependent cell types (*26*). However, the increased resolution of our dataset raised the possibility of identifying more specialized cell types that could be size dependent. For instance, larger organisms may have specialized energy storage types. Furthermore, processes such as sexualization are known to lead to the formation of new organs (e.g., gonads and reproductive apparatuses). Therefore, to determine if there are body size–specific cell types, we assessed if all detected cell populations were present across all body sizes.

**Fig. 3.**
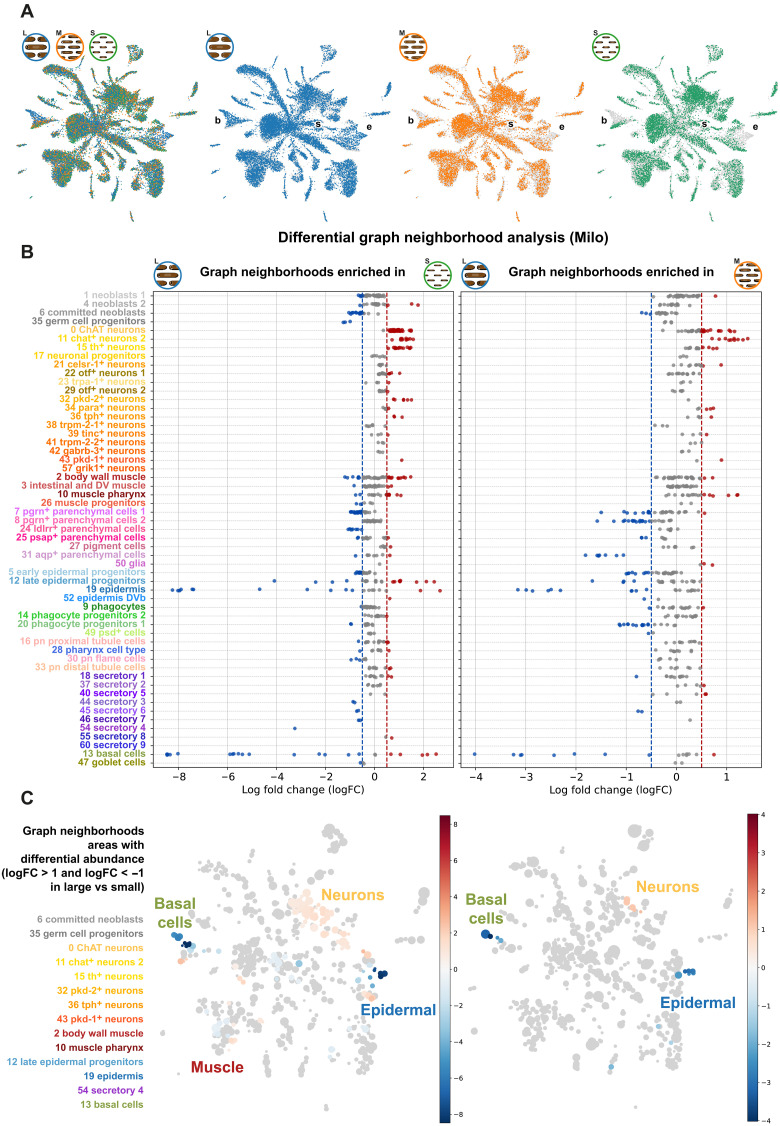
Neighborhood-based cell-type differential abundance testing in planarians of different sizes. (**A**) UMAP of L, M, and S cells in the UMAP space merged and by individual body size category. b: basal cells, e: epidermal cells, s: secretory cells. (**B**) Differential graph neighborhood analysis (Milo). Log fold changes for each graph neighborhood are represented and classified by cluster of origin, for the L versus S (left) and L versus M (right) comparisons. (**C**) UMAP embedding of graph neighborhoods with color-coded log fold changes. Clusters with neighborhoods with high log fold changes are highlighted in the left.

We first examined qualitatively the distribution of L, M, and S cells in the UMAP space. Interpreting UMAPs in such a way is controversial as dimensionality reduction techniques and low-dimensional embedding of single-cell data are known to introduce distortions of the dataset (*53*). On the other hand, our multiplexed analysis to minimize batch effects allows us to expect a homogeneous distribution of all three samples, except for biological differences. In our dataset, L, M, and S cells were homogeneously distributed throughout the UMAP except in regions corresponding to the basal cells (cluster 13), the epidermis (cluster 19), and the secretory 4 (cluster 54) (Fig. 3A). To investigate if these effects were based on biological differences or stochastic variation due to dimensionality reduction, we explored the parameter space of our principal components analysis (PCA), *k*-nearest neighbor (kNN) embedding and UMAP (fig. S3). This analysis revealed that UMAP differences in basal cells were observable in all analyses with at least 55 principal components, and despite the kNN parameter. This strongly suggests that the differences in the basal cell cluster are of biological source and motivated further inquiry.

We then wondered if planarians of different body size contain cell states that are differentially enriched. To address this question, we used Milo (*54*), a differential abundance testing statistical framework that exploits kNN graphs to assign cells to neighborhoods. Milo then tests the differential abundance of each condition in these neighborhoods, which can be then associated with different cell states.

We used Milo to perform L versus S as well as L versus M comparisons (Fig. 3B). Milo revealed overall stronger effects on the former comparison, consistent with our expectations. Many cell clusters had neighborhoods of enriched or depleted abundance in L versus S or medium samples, but the overall strongest effects were present in the late epidermal progenitors, epidermis, and basal cell populations. We then plotted these neighborhoods on the UMAP embedding (Fig. 3C), revealing that these neighborhoods largely correspond to the areas highlighted in the UMAP (Fig. 3A). In particular, the planarian epidermis and the basal cells contain neighborhoods enriched in both L and S planarians. This suggests that there are cell states that are differentially abundant in planarians of different size.

### Asexual planarians of different sizes are made of the same cell types

We then asked if these differences translated into body size category-specific cell clusters. To decouple this analysis from the resolution parameter of the cell clustering algorithm, we performed this analysis using four different resolutions of the Leiden algorithm (1 to 4) (fig. S4, A to D). This analysis showed that all three size categories have cells from all clusters except for the rarest clusters, i.e., cluster 64 of resolution 3 (11 cells; fig. S4C) and cluster 72 of resolution 4 (eight cells; fig. S4D). This strongly suggests that this is a sampling effect due to the low cell numbers of these clusters. Further to that, cluster 54 of resolution 3 and clusters 43 and 57 of resolution 4 are largely made of L and M cells (fig. S4, C and D), with only a few S cells (one in each case). These clusters correspond to the differences observed before on UMAP space. Cells from cluster 19 of resolution 3, corresponding to the epidermis (fig. S4C), are subclustered in two clusters of resolution 4, clusters 29 and 43 (fig. S4D). The latter cluster was mostly made of cells from the L planarian sample (125 L, 12 M, and 1 S) and expresses markers of both epidermis and late epidermal progenitors (data S4). Cluster 13 of resolution 3, containing the basal cells (fig. S4C), remains one cluster (*14*) at resolution 4 (fig. S4D). Therefore, we argue that the basal cells are one cell type, and the differences observed in UMAP space correspond to differences in the state of basal cells. Overall, this analysis shows that asexual planarians of our three size categories are made of the same essential cell clusters but that different cell states are differentially abundant in each size category. Furthermore, differences in cell-type abundance may exist.

### Planarians of different body sizes have different cell-type proportions

**Fig. 4.**
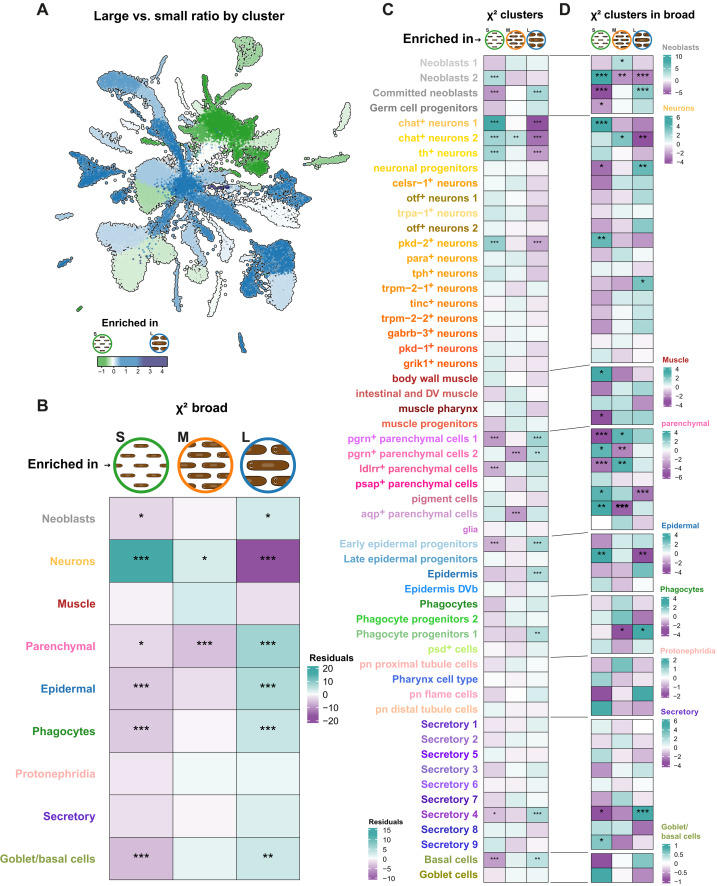
Differences in cell proportions in planarians of distinct sizes. (**A**) UMAP visualizing the percentage log_2_ ratios of cell clusters in L versus S planarians. (**B**) Heatmap representation of the results of the chi-square test examining the association between S, M, and L in broad groups. (**C**) Heatmap representation of the results of the chi-square test examining the association between S, M, and L in the 55 annotated clusters. (**D**) Heatmap representation of the results of the chi-square test examining the association between S, M, and L in each cluster of the broad group. Color intensity represents the residuals (i.e., the differences between the observed and expected frequencies in each cell of the contingency table) from the chi-square test; green tones indicate increased frequency of cell types (surplus of observed counts in that category), and purple tones indicate decreased frequency of cell types (deficit in observed counts in that category). Significance levels are denoted by asterisks (*) based on the Benjamini-Hochberg–adjusted *P* values. *P < 0.05, **P < 0.01, and ***P < 0.001.

We then analyzed the differences in cell-type frequency at the cluster level among L, M, and S planarians. To visualize these differences in the UMAP space, we plotted the percentage ratios of each cell cluster in L versus S planarians (Fig. 4A). The values examined correspond to the logarithmic ratio between the percentage of each cluster in L planarian samples versus S planarian samples. In this representation, a log_2_ ratio of 1 indicates that the cell cluster percentage is double in L than in S planarian samples.

We analyzed the frequencies using a chi-squared test with a Benjamini-Hochberg *P* value adjustment (data S7). Each test compares cells within a size category and broad cell type to those in other categories and cell types, generating contingency tables that contrast cells of a given broad type in one size category with cells of the same type in other categories and cells of other types within and outside the size class.

We examined the power of the chi-squared test (*55*), taking into consideration the cell sample obtained in our experiment (Fig. 2A) and the frequencies of individual cell populations in planarian samples (Fig. 2D), determining that chi-squared tests can find significant effects of ±2% with statistical power across the range of frequencies (note S1 and fig. S5). To elucidate if our results compare well with those published by microscopical observation, we first analyzed the dataset grouped by broad-type category. This grouping made our categories largely comparable with those analyzed by Baguñà and Romero (*26*). Our analysis revealed an increased frequency of neurons in S planarians and a decrease in L planarians (Fig. 4B), consistent with the microscopy findings. We observed significantly increased frequencies in L and decreased in S for the neoblasts, parenchymal, epidermal, phagocytes, and goblet/basal cell broad categories (Fig. 4B). The increases in parenchymal, phagocytes, and goblet/basal are also consistent with the reported increases of fixed parenchymal cells, gastrodermal, and goblet cells in the microscopy data. Our analysis revealed tendencies inconsistent with those reported by Baguñà and Romero (*26*) for the neoblast and epidermis broad groups, which were described to be more frequent in S planarians.

Next, to exploit the increased cluster resolution capacity of single-cell transcriptomics, we repeated the chi-squared analysis in our 55 annotated clusters (Fig. 4C). This analysis revealed that the clusters neoblasts 2 and committed neoblasts showed opposite trends, with the neoblasts 2 overrepresented in S planarians and the committed neoblasts underrepresented in S planarians and overrepresented in L planarians instead. This can potentially explain the discrepancy with the microscopy dataset, as this dataset did not include progenitor cells. It is unclear how this study classified cells that have started to differentiate, but likely these cells already displayed differentiation markers and were classified within their differentiated cell-type category. Next, we examined the individual neuron clusters. We found significant overrepresentation in S and underrepresentation in L planarians for the *chat*^+^ neurons clusters 1 and 2, the *th*^+^ neurons and the *pkd*-*2*^+^ neurons. Most other neuron clusters had similar tendencies but were not significant in this analysis. Among the parenchymal broad group, we found that the *pgrn*^+^ clusters 1 and 2 were significantly enriched in L planarians and significantly underrepresented in S and M planarians. Furthermore, the *ldlrr*^+^ cells were also found significantly reduced in S planarians. The *aqp*^+^ cells were overrepresented in M planarians and was the only cluster with this behavior. The biology and function of the parenchymal cells are still enigmatic, and the biological significance of this observation is unclear. From the epidermal cells, we found significant enrichment in L planarians for the early epidermal progenitors and the epidermis. We also found a significant enrichment of phagocyte progenitors 1, secretory 4 cells, and the basal cells. Together, this analysis shows that single-cell transcriptomics has higher resolution and is able to detect significant differences at the cell cluster level, beyond the broad-type categories.

Next, we analyzed each of these clusters compared to their broad category (Fig. 4D). This analysis aimed to identify cell types that behave contrary to their broad type. Our results corroborated the contrary tendencies of neoblasts 2 and committed neoblasts. We also observed that *trpm*-2-2^+^ neurons were enriched in the neuronal subgroup of L planarians, a trend opposite to that of neurons as a group. Other clusters behaved similarly but were not significant. Notably, the neuronal progenitors also have a significant enrichment in the neuronal broad group of L planarians. This analysis also revealed a significant enrichment of body wall muscle in S planarians when compared to the muscle broad group. Within the parenchymal broad group, we found highly significant underrepresentation of *pgrn*^+^ and *ldlrr*^+^ cells in S planarians and underrepresentation of pigment cells in L planarians. Collectively, these results show that studying allometry with single-cell transcriptomics has a high resolution and identifies several cell types that behave contrary to their broad-type categories.

### Head cell types are enriched in small planarians, and intestinal and parenchymal cell types are enriched in large planarians

**Fig. 5.**
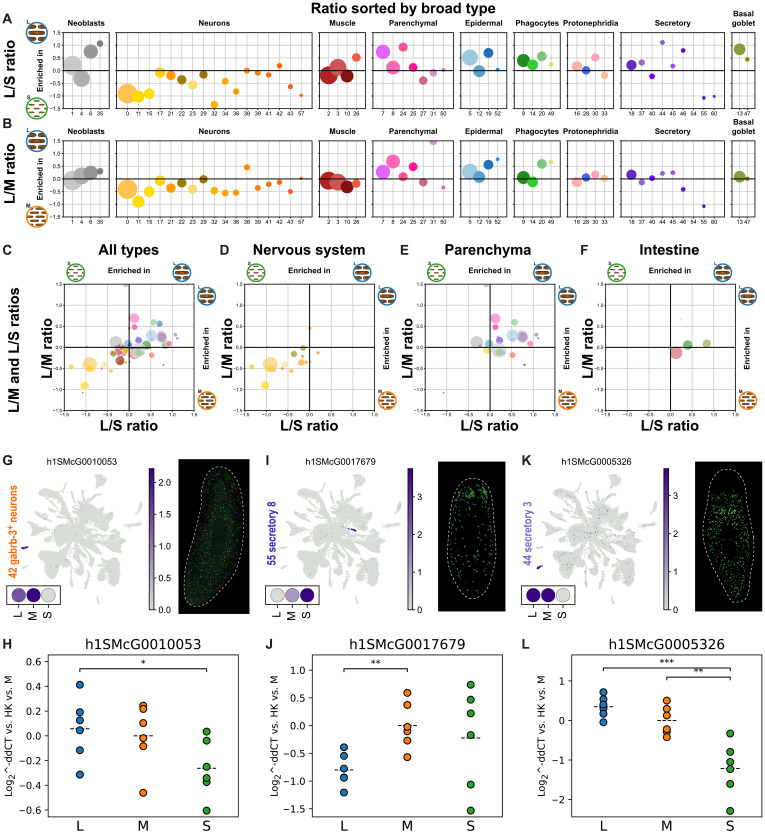
Variations in cell proportions by anatomical features. (**A**) Percentage ratios of cell clusters in L versus S planarians sorted by broad group. (**B**) Percentage ratios of cell clusters in L versus M planarians sorted by broad group. In both plots cluster 54, corresponding to secretory 4 cells, lies outside of the plotted area due to a high enrichment in L, and cluster 60, corresponding to secretory 9 cells, lies outside of the plotted area due to a high enrichment in M. (**C** to **F**) Comparison of L versus S and L versus M ratios in all types (C), nervous system types (D), parenchymal types (E), and intestine types (F). In all plots, dot size represents cluster size in cell number. (**G**, **I**, and **K**) Feature UMAP plot, dotplot, and in situ picture of marker genes of *gabrb*–*3*^+^ neurons [cluster 42 (G)], secretory 8 [cluster 55 (I)] and secretory 3 [cluster 44 (K)]. All in situ pictures retrieved from the database digiworm (https://digiworm.wi.mit.edu/). (**H**, **J**, and **L**) qPCR validation of the same markers in L, M and S planarian RNA samples. Significance levels are denoted by asterisks (*) based on two-sided *t* tests: *P < 0.05, **P < 0.01, and ***P < 0.001.

We then tested if the patterns observed could be explained by anatomical features. To investigate this, we examined the percentage ratios of each cell cluster in L versus S planarians (Fig. 5A) and L versus M planarians (Fig. 5B) sorted by broad type. This analysis revealed that neuronal types are generally enriched in planarians of the smaller size, and parenchymal types are enriched in L planarians instead. The values of both L versus S and L versus M are well correlated (Fig. 5C). We then assigned cell clusters to anatomical features based on previous literature (data S2 and fig. S6). In brief, “body wall” corresponded to body wall muscle (*56*), pigment cells (*57*–*59*), protonephridia distal tubule cells (*36*, *37*, *60*), and the epidermis (*47*, *61*). “Intestine” cell types (*39*, *41*, *62*) included phagocytes, goblet cells, basal cells (*39*), *psd*^+^ cells (*44*), and intestinal muscle (*56*). All neuronal types and the cell cluster described as glia (*63*, *64*) were classified as “nervous system.” “Pharynx” contains the pharynx cell type (*44*) and the muscle of the pharynx (*44*). All other cell clusters were considered part of the “parenchyma,” i.e. the space between the body wall and the intestine. This included parenchymal cells (excluding pigment and glia, located in the body wall and the nervous system), secretory cell types, neoblasts, and progenitor clusters. This analysis revealed that body regions largely explain the observed frequencies, with nervous system cell types being broadly enriched in S and M planarians (Fig. 5D) and intestinal and parenchymal cell types being broadly enriched in L planarians (Fig. 5, E and F).

To orthogonally validate these observations, we measured by quantitative polymerase chain reaction (qPCR) the differential expression of markers specific to these cell types in newly generated independent RNA samples (data S8). We repeated the animal classification in sizes L, M, and S, then extracted RNA, and generated cDNA from three biological replicates of each size category.

We first focused on neuronal markers. We validated by qPCR the enrichment of several neuronal clusters in RNA samples from S planarians and the depletion in L planarians (fig. S8A). We then reasoned that our scRNA-seq experiments had increased cell-type resolution compared to previous studies. For instance, not all neuronal types must be scaling at the same rate. To gain more insight into this notion we examined the most extreme values of our L versus S ratio and sought to validate these observations by querying previously published in situ hybridization experiments (*42*) and by performing qPCR experiments. Cluster 42 cells were the nervous system cell cluster with the highest L to S ratio, a pattern dissimilar to other neuronal cell types (Fig. 5G). Cluster 42 cells were not enriched in heads but are distributed throughout the body (Fig. 5G). We validated this observation by qPCR (Fig. 5H).

We then focused on secretory cell clusters. We first analyzed markers of cluster 55 as they had the lowest enrichment in L planarian samples (Fig. 5B). Their distribution showed accumulation in head regions (Fig. 5I), explaining the enrichment in S planarian samples, similar to that of neuron types. We validated the depletion of this marker (Fig. 5J) and an additional marker (fig. S8B) in L planarian samples by qPCR. We then examined a marker of cluster 54 (secretory 8) since it had the highest value of L/S ratio. Our qPCR experiments showed agreement with the single-cell data, validating the enrichment in L and depletion in S planarian samples (fig. S8B). Cluster 44 secretory cells had also strong enrichment in L planarian samples (Fig. 5B). Consistently, we observed that these cells are broadly distributed throughout the parenchyma but depleted from the head region (Fig. 5K). This enrichment in L planarian samples was also validated by qPCR (Fig. 5L). Together, these analyses show that S planarians have comparatively more head cell types, including nervous and secretory types, and L planarians instead quantitatively contain more parenchymal and intestinal cell types.

### Small planarians have increased body wall and body margin cell types

**Fig. 6.**
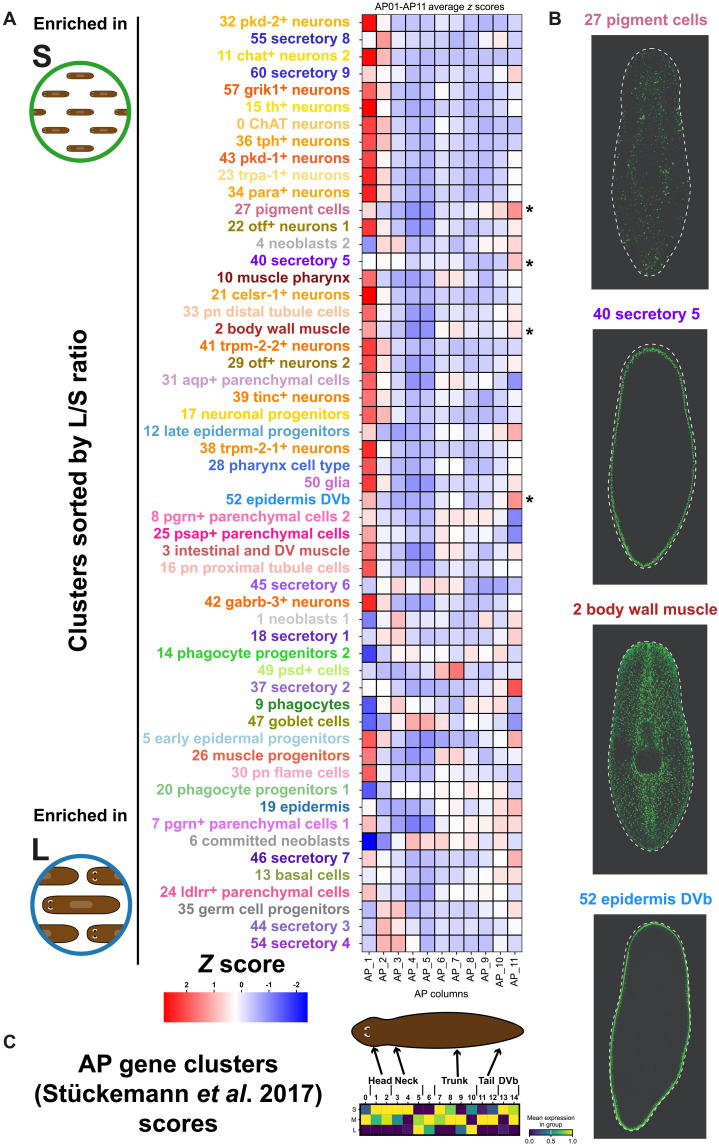
Comparison of large versus small enriched clusters with anterior-posterior enrichement. (**A**) Heatmap of *z* scores showing the enrichment of cluster markers sorted by L versus S enrichment in anterior-posterior samples AP1 to AP11 from Stückemann *et al*. (*65*). Clusters with high *z* scores in both AP1 and AP11 are indicated with an asterisk. (**B**) In situ picture of marker genes of clusters with high *z* scores in AP1 and AP11. All in situ pictures retrieved from the database digiworm (https://digiworm.wi.mit.edu/). (**C**) Scores of AP gene cluster genes described by Stückemann *et al.* (*65*) in L, M, and S samples and cartoon indicating the enrichment in body regions described in the original publication.

We sought to obtain additional validation by comparing our results to those of Stückemann *et al*. (*65*). They performed bulk RNA-seq experiments on planarian tissue samples obtained along the anterior-posterior axis. First, we calculated an average *z* score for the top markers in our single-cell clusters, assessing their expression across the anterior-posterior dataset (Fig. 6A). Supporting our conclusions, cell types with high enrichment in L planarian samples displayed high *z* scores in anterior-posterior fragments AP1 and AP2 (of 11 total fragments), corresponding to the “head” regions as defined by Stückemann and colleagues (*65*). This includes neuronal types and non-neuronal types, such as cluster 55 (secretory 8) and parenchymal cluster 50 (glia). These findings largely confirm the results presented in Figs. 4 and 5, strengthening our conclusion that smaller planarians are enriched in head cell types, both neuronal and non-neuronal.

However, this analysis uncovered additional patterns. Some clusters, despite showing high enrichment in S planarian samples compared to L, did not exhibit high *z* scores in the anterior fragments identified by Stückemann *et al.* (*65*). Instead, these clusters showed high scores in the most posterior fragment (AP11) or presented a mixed pattern with enrichment in both anterior and posterior fragments. These clusters correspond to cell types typically located in the body wall, including pigment cells, specific secretory cell types, and epidermal cells in the dorso-ventral boundary (DVb) (Fig. 6B).

Last, we conducted a reverse analysis by retrieving markers from the clusters described by Stückemann *et al*. (*65*) and scoring their expression in our dataset. Clusters 1 to 5, identified by Stückemann *et al*. (*65*) as enriched in the head and neck regions of planarians, were similarly enriched in our S planarian samples (Fig. 6C). In addition, clusters 13 and 14, enriched in the DVb, also showed a consistent enrichment in S planarian samples (Fig. 6C). These analyses confirm the enrichment of head types in S planarians and reveal a similar enrichment of body wall and margin.

### Differential gene expression analysis reveals cell type–specific size-related gene modules

**Fig. 7.**
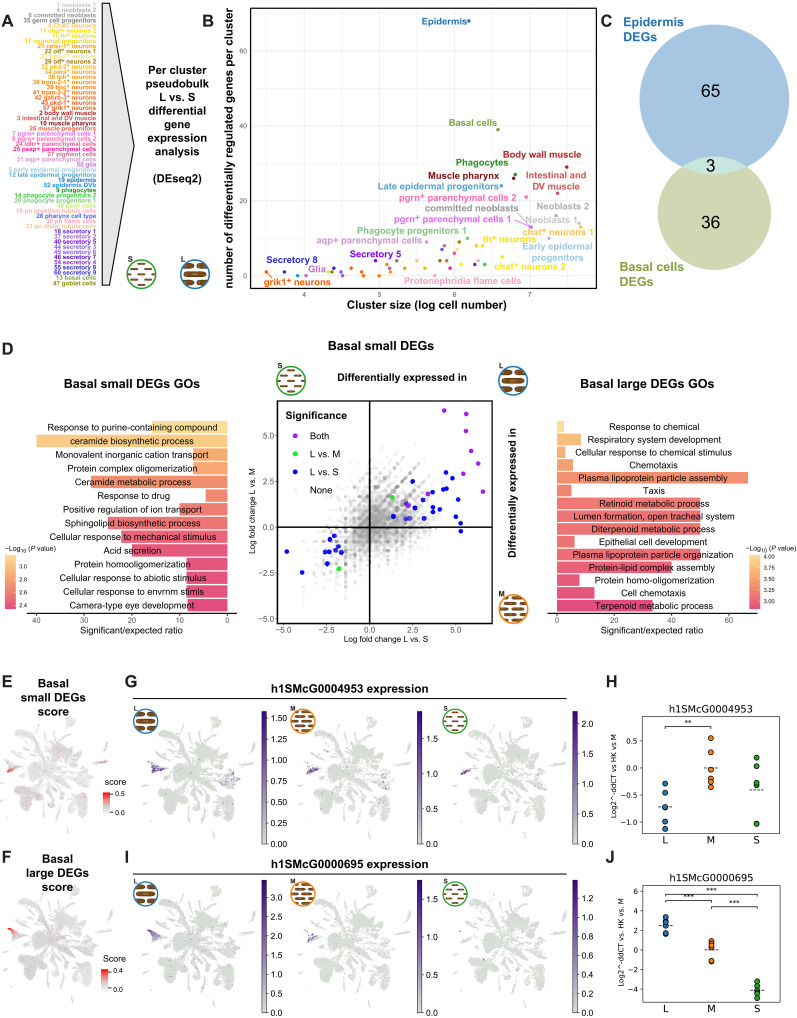
Size-dependent cell type–specific gene modules in L and S planarians. (**A**) Generation of pseudo-bulk count tables for the 55 annotated cell clusters. (**B**) Differential gene expression analysis with DEseq2. Scatter plot showing the number of differentially regulated genes per cluster compared to the log-transformed cell count within each cluster. (**C**) Overlap between differentially expressed genes detected in epidermis and basal cells. (**D**) Scatter plot of differential gene expression analysis comparing L versus S and L versus M log fold changes in computationally dissected basal cells and GO term enrichment analysis of significant genes in the L versus M comparison. Bar size indicates ratio of significant/expected fraction of annotated genes with a given GO term. Color gradient indicates adjusted *P* value (Fisher’s exact test). (**E** and **F**) UMAP visualization of scored expression of basal cell differentially regulated genes. (**G** and **I**) UMAP features plots of differentially regulated genes, sliced in L, M, and S samples. (**H** and **J**) qPCR validation of the same markers. Significance levels are denoted by asterisks (*) based on two-sided *t* tests: **P < 0.01, and ***P < 0.001.

We then aimed at determining if cell types change their gene expression patterns in response to animal size. For this analysis, we focused on L and S planarian samples. Taking full advantage of our multiplexed single-cell approach, we aggregated cluster information to generate pseudobulk (*66*–*68*) count tables for each specific cluster (Fig. 7A). We used the two libraries of the experiment (Fig. 1A) as pseudoreplicates. The two libraries come from the same experiment and can only be considered technical replicates of the tagmentation and fourth barcoding step. However, we reasoned that these are indeed made of completely different cells. It has been recently shown that pseudoreplication (i.e., computationally distributing cells in random groups to obtain replicate information) works well for single-cell differential gene expression analysis (*69*). Arguably, this is because in single-cell approaches, there is an additional level of biological replication in the single-cell barcoding. We analyzed the 65 independent cluster-aggregated count tables using DEseq2 (*66*) to identify genes differentially regulated by size (data S9).

To elucidate if there are cell types that respond more dynamically to size differences, we examined the relationship between cluster size and the number of differential genes detected (Fig. 7B) in each cell cluster. The number of differential genes increased with cluster size because this is correlated with the number of reads that go into the analysis, maximizing its power. However, two clusters lie outside of this distribution, with a much higher number of differentially regulated genes: the epidermis (cluster 19) and the basal cells (cluster 13). These are the clusters identified in the UMAP analysis, likely explaining the UMAP differences. These lists overlapped by only three genes (Fig. 7C), indicating that the differences in gene expression of epidermal and basal cells are largely independent and cell type specific. This analysis shows that the epidermis and the basal cells are the planarian cell types that respond more dynamically to animal size at the gene expression level, with largely independent gene modules.

To examine the genes that are differentially regulated in the epidermis, we obtained volcano plots and analyzed the Gene Ontology (GO) term enrichment of up- and down-regulated genes (fig. S9). Genes differentially expressed in epidermal cells from S animals were enriched in cilium and other related GOs (fig. S9). Genes differentially expressed in epidermal cells of L animals were enriched in creatine metabolism and other metabolic processes, suggesting that in L planarians, epidermal cells carry out metabolic functions (fig. S9). Key genes in this enrichment are AGAT enzymes (*h1SMcG0009479*, *h1SMcG0009480*, and *h1SMcG0011148*), which have been already described in planarian late epidermal progenitors (*42*, *44*, *45*, *47*, *70*).

We then focused on the recently described basal cells (*39*). They form a cluster (*13*) that does not separate in any of the clustering resolutions tested (fig. S4). However, graph neighborhood differential abundance showed individual graph areas enriched in S and L planarians (Fig. 3). Differential abundance at the cluster level (Fig. 4 and 5) showed the basal cell cluster is enriched in L planarians. This suggested that basal cells exist in two different cell states, one enriched in L planarians and another one enriched in S planarians.

To further investigate this question, we studied the differential expression between L, M, and S planarians in computationally dissected basal cells (Fig. 7E). Most significant changes corresponded to genes up-regulated in L animal basal cells. The L versus S comparison had more significantly regulated genes (*39*) compared to the L versus M (*11*), but many genes overlapped (*9*). Genes differentially expressed in basal cells of the S planarian sample were enriched in ceramide sphingolipid biosynthesis genes (Fig. 7D). Genes differentially expressed in basal cells of L animals were enriched in lipoprotein particle assembly, retinoid, and terpenoid metabolism genes (Fig. 7D). To elucidate if these genes are exclusively expressed in the basal cell cluster or, if contrarily, they are expressed more generally in other clusters as well, we examined their UMAP feature plots (fig. S7). We found that the differentially expressed genes are expressed in other cell types. We then scored the expression of these gene sets to obtain a general view of their grouped expression. Genes differentially regulated in basal cells from S animals and L animals were expressed in graph neighborhood areas more abundant in the S planarian sample and in the L planarian sample, respectively (Figs. 7, E and F and 3). This is consistent with the idea of two basal cell states. We examined the expression of one gene down-regulated in both differential gene expression comparisons (*h1SMcG0004953*; Fig. 7G), revealing that all three size categories expressed the gene. We performed qPCR analyses and found that this gene was only significantly down-regulated in L planarians compared to M (Fig. 7H), and the gene was not significantly down-regulated in the differential gene expression analysis either (Fig. 7D). When we checked a gene that was significant in both L versus S and L versus M comparisons (*h1SMcG0000695*; Fig. 7I), we found that this gene was indeed present in the graph neighborhood area enriched in L basal cells and that the gene (and the graph neighborhoods) were nearly absent from the S planarian sample. We perform qPCR analyses to confirm these results and found significant differences in expression in all comparisons. This shows that planarian basal cells are present in two cell states; one is present in planarians of all sizes but differentially enriched in S planarians (Fig. 4), and the other is differentially enriched in L planarians and nearly absent from S planarians (Figs. 4 and 7I). We hypothesize that these cells are responsible for the accumulation of lipids around the basal area of the gut described previously (*27*).

## DISCUSSION

Cell-type allometry is a key question in developmental biology, but it has remained mysterious in many organisms due to the lack of cell-type markers (antibodies, RNA probes). Furthermore, cell quantification with methods of immunohistochemistry and/or in situ hybridization is challenging at full body scale. Cell-type identification based on microscopic observation presents a subjective component. Last, these methods can only identify a few cell types and lack resolution.

Here, we showed that single-cell transcriptomics solves these challenges by providing a method to identify and quantify all major cell types in different samples. Beyond that, scRNA-seq allows accessing the gene expression patterns of each cell type. To achieve this level of resolution, it is key to use fixation methods such as ACME (*21*) and multiplexing approaches such as SPLiT-seq (*22*). These methods will open the door to studying cell-type allometry and other cell-type quantitative studies in virtually every organism.

**Fig. 8.**
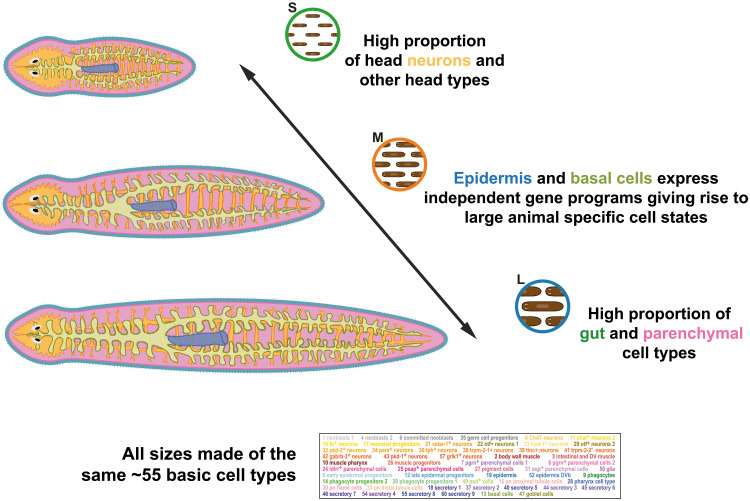
Graphical representation of the cellular and molecular basis for cell-type allometry in planarians. Representation of the key allometric variation depicted via single-cell combinatorial barcoding approach. Presence of the ~55 basic cell types in all planarian sizes. High proportion of neurons of the head region and other non-neuronal head cell types in S planarians. Independent gene modules in epidermis and basal cells give rise to L planarian–specific cell states. High proportion of gut and parenchymal cell types in L planarians.

Planarians are an excellent model to study cell-type allometry because of their ability to grow and degrow with food availability. Previous studies have already shown that neurons are enriched in small planarians and that gut and parenchymal cell types are enriched in large planarians. Our study confirmed these trends but provided higher resolution. For instance, we were able to identify neuron types that are not enriched in S planarians and secretory types enriched in heads. Our results show that the ratios for each cell type are largely explained by their anatomical location despite their broad cell-type identity, with S planarians having an enrichment of head cell types (Fig. 8).

Baguñà and Romero (*26*) showed that asexual planarians were made of the same basic 13 broad cell types in varying proportions. We confirm these results, but at a higher resolution of ~55 annotated cell clusters. Single-cell transcriptomics is changing the way we think about cell types, showing that granularity is key to their definition (*9*). We were able to show that there are no cell clusters specific to large or small asexual planarians at our current level of resolution. We were able to distinguish up to 17 types of neurons, 7 types of parenchymal cells, and 9 types of secretory cells and found them all represented in all size categories. While further subdivision of these types might lead to the identification of types specific to small or large planarians in the future, the question would then be if these correspond to cell types or if they constitute different cell states of the same specific cell types.

We identify cell states characteristic of large planarians for the epidermis and the basal cells. We argue that these represent states instead of types because (i) they are just defined by a few quantitative differences in gene expression and (ii) they share general markers of their cell type. Increasing the resolution of the Leiden clustering algorithm can cluster these cells out, but at these high resolutions, other cell types are divided in clusters that lack meaningful markers and are largely explained by noise. Our results delve further into the definitions of cell type and cell state and will offer insight to other researchers investigating the nature of cell-type identity.

Crucially, we are able to access the gene expression patterns of these cell types and states and identify differentially regulated genes. Further studies will identify more gene expression differences, enabled by deeper resolutions, larger cell numbers, and increased number of samples and replicates. This will allow us to study their transcriptional regulation, including the transcription factors that regulate the differences. Combinatorial barcoding scRNA-seq methods such as SPLiT-seq and other single-cell analysis methods will be key to decode the cellular and molecular principles of cell-type allometry and other developmental paradigms.

Our results are largely consistent with those of Baguñà and Romero’s microscopic study, except for two key broad groups: the neoblasts and the epidermis. Key to understanding this discrepancy is the fact that the microscopy study did not identify progenitor cells. We were able to identify seven progenitor clusters, the majority of which are enriched in large planarian samples. This also applies to the committed neoblast cluster. It is unclear whether Baguñà and Romero (*26*) classified progenitor cells with neoblasts or with their corresponding cell fate broad group. The overrepresentation of progenitors in large planarians raises interesting hypotheses about the dynamics of cellular differentiation. For instance, the differentiated epidermis contains a group of cells that clusters together with the mature epidermis but contains late epidermal progenitor cell markers and is highly enriched in large planarians. This could indicate a longer time of differentiation for epidermal cells in large planarians. Future research will address the dynamics of cell differentiation in planarians and other organisms using single-cell transcriptomics.

The most dynamic cell type in planarians of different sizes are the basal cells, as they change both their frequency and their gene expression patterns. This type was only described recently (*39*) with proposed metabolic functions. In previous single-cell transcriptomic dataset, the basal cells are found clustered together with other gut cell types (*44*), primarily goblet cells (*21*), due to their similarity. We hypothesize that the basal cells host the size-dependent lipid accumulations described by Thomenn *et al*. (*27*). Our data show that basal cells exist in two states characterized by their gene expression patterns. Basal cells in large animals express lipid particle synthesis and assembly genes. Our results will open the door to further studies about the biology of this enigmatic planarian cell type.

## MATERIALS AND METHODS

### Planarian culture and maintenance

The experiments were conducted using the asexual strain derived from the clonal line Berlin-1 of *S. mediterranea*. The animals were kept at a temperature of 20°C in 1× Montjuic water (*71*), which consists of 1.6 mM NaCl, 1.0 mM CaCl_2_, 1.0 mM MgSO_4_, 0.1 mM MgCl_2_, 0.1 mM KCl, and 1.2 mM NaHCO_3_ dissolved in deionized water. The pH of the solution was adjusted to approximately 7.5 using 1 M HCl. The planarians were fed fresh cow liver every 4 to 5 days.

### Sized animal selection and photography

Animals encompassing all sizes in our asexual cultures were taken from boxes that had not been feed for 4 to 10 days. These animals were pooled together in the same containers to introduce a level of randomization, and they were starved for further 3 days, ensuring that all animals had 1 to 2 weeks of starvation before the experiment. On the day of the experiment, we separated animals that had fissioned in the last 3 days. Then, the animals were manually selected at random and classified based on their sizes into different experimental groups through visual assessment. Our primary objective was to categorize the animals into four distinct body sizes, specifically designated as extra large (XL), L, M, and S. To ensure that cell numbers are comparable, we selected 10, 25, 50, and 100 animals for the XL, L, M, and S size categories respectively. After animal selection, animals were photographed, and ACME was dissociated on the same day to avoid changes in size (e.g., by fission).

To obtain visual records, we took photographs, with a standardized methodology of capturing groups of five animals per picture on an A4 graph millimeter paper. The process avoided animal manipulation under scope bright light to avoid inducing stress. We captured low-resolution images using a Lumens PC193 standard field camera, using the default settings and a 35-mm focal length. We proceeded to measure the area of the individual animals to ensure the accuracy of our examination and quantify the size separation range. The images were processed and analyzed using ImageJ 1.51d software, treating them as 8-bit pictures, enabling us to measure the animal’s area in pixels. Subsequently, we calculated the corresponding area in square millimeter by comparing the measurements of animal pixels with the known measurements of the pixel scale present on the millimeter paper within each picture.

### ACME dissociation

The ACME solution was freshly prepared by combining commercially sourced deoxyribonuclease/ribonuclease-free distilled water, methanol, glacial acetic acid, and glycerol in a ratio of 13:3:2:2 (*21*). Each sample was placed in a 15-ml Falcon tube. Montjuic water was removed using a Pasteur pipette, and approximately 100 to 500 μl of 7.5% N-acetyl cysteine in 1× phosphate-buffered saline (PBS) was added to cover the planarians and remove the mucus and protect the RNA. The ACME solution was promptly added to each sample, reaching a final volume of 10 ml per tube. The samples were then left to dissociate at room temperature for 45 min on a see-saw motion shaker at 35 to 45 rpm, with tubes oriented parallel to the direction of movement. We complete dissociation mechanically by pipetting the reactions up and down using 1-ml low-binding pipette tips. From this point, the samples were kept on ice to prevent RNA degradation.

To remove cell aggregates and undissociated tissue fragments, each sample was filtered through a 50-μm Celltrics filter into a new 15-ml tube and then subsequently filtered through a 40-μm Celltrics filter into another tube. The sample was centrifuged at 1000*g* for 5 min (4°C), discarding the supernatant except for 1 to 2 ml, which was used to resuspend the pellet. We filter each sample one more time using 40-μm cell strainer pipette tips (1000 μl) into a new 15-ml tube.

To clean the cells, 7 ml of buffer [1× PBS and 1% bovine serum albumin (BSA)] was added to each sample, followed by centrifugation at 1000*g* for 5 min (4°C). The supernatant was removed, and the pellets were resuspended in 900 μl of buffer (1× PBS and 1% BSA) and transferred to 1.5-ml Eppendorf tubes. For cryopreservation, 100 μl of dimethyl sulfoxide was added to each tube (*72*), and the samples were directly stored at −80°C.

### SPLiT-seq

scRNA-seq libraries were generated using a combinatorial barcoding approach, following a customized adaptation of the SPLiT-seq protocol (*22*). We developed and refined a 4-day protocol, incorporating various optimizations, to achieve improved efficiency and reliability.

#### Day 1: Rounds 1, 2, and 3 of DNA barcoding

For cytoplasm staining, the dissociated samples were labeled with a concentration of 2 μl/ml from a stock solution (1 mg/ml) of Alexa Fluor 488–conjugated concanavalin A (Invitrogen). In addition, for nuclear staining, a concentration of 1 μl/ml from a 5 mM stock solution of Draq5 (eBioscience) was used. The labeling process took place in the dark at 4°C for a duration of 30 to 45 minutes. Subsequently, the samples were visualized using a CytoFlex S Flow Cytometer (Beckman Coulter) to determine the count of single cells per sample and measure the percentage of cells in G_1_ and G_2_ phases.

The first round of barcoding was carried out through in-cell reverse transcription (RT). Anchored oligo (dT) oligos were used to minimize the number of ribosomal reads. Each reaction used ~5000 singlet events as calculated from the flow cytometry count in a volume of 8 μl per well. Following this, a second and third round of barcoding were performed using ligation reactions (*21*).

#### Day 2: FACS sorting and cell lysis

Following the barcoding process, cells were sorted using a BD FACS Aria III Cell Sorter (BD Biosciences) in 50 μl of lysis buffer [comprising 20 mM tris (pH 8.0), 400 mM NaCl, 100 mM EDTA (pH 8.0), and 4.4% SDS]. A maximum of 25,000 events were sorted into each tube. To ensure the absence of RNase contamination, the FACS instrument was meticulously cleaned with bleach and precooled before sorting. The injection and collection chambers were maintained at 4°C throughout the sorting process. Sorting was carried out using the BD FACSDiva Software, set up in a four-way purity mode, with an 85-μm nozzle and moderate-pressure separation (45 Psi).

Subsequently, the volume of each sorted sublibrary was adjusted to 100 μl, and 10 μl of Proteinase K (20 mg/ml) was added to each tube. The lysates were then incubated at 55°C for 2 hours, with manual shaking of the tubes every 15 min. Last, the lysates were stored at −80°C for further use.

#### Day 3: cDNA purification, template switch, and dsDNA amplification

To purify the cDNA from genomic DNA, we used 44 μl of Dynabeads MyOne streptavidin C1 (Invitrogen) per lysate. These magnetic beads were conjugated with streptavidin, which binds to the biotin present at the 3′-end of the third barcode. The manufacturer’s protocol for Dynabeads nucleic acid purification was strictly followed without any deviations.

Next, the cDNA was converted from single-stranded DNA (ssDNA) to double-stranded DNA (dsDNA) through a template-switch reaction. This reaction involved the following components per sample: 44 μl of 5× RT buffer (Thermo Fisher Scientific), 44 μl of 20% Ficoll PM 400 (Sigma-Aldrich), 22 μl of a mixture containing 10 mM of each of the four deoxynucleotide triphosphates (NEB), 5.5 μl of Template Switch Oligo (100 μM), 11 μl of Maxima H Minus RT, and 88 μl of nuclease-free water. The samples were incubated in this reaction mix for 30 min at room temperature, followed by 90 min at 42°C with agitation. After incubation, the template switch mix was removed using a magnetic rack.

For cDNA amplification, a total of 121 μl of 2× Kapa HiFi HotStart ReadyMix (Roche), 9.68 μl of PCR_PF (10 μM), 9.68 μl of PCR_PR (10 μM), and 101.64 μl of nuclease-free water were added to the cDNA samples. Each sample was resuspended in 220 μl of the PCR mix and divided into four PCR tubes. The following thermal cycling program was run: an initial denaturation step at 95°C for 3 min, followed by five cycles of denaturation at 98°C for 20 s, annealing at 65°C for 45 s, and extension at 72°C for 3 min. The four PCR reactions were then combined into a single 1.5-ml Eppendorf tube, and the Dynabeads were separated using a magnetic rack. Two hundred microliters of the supernatant, containing the cDNA in suspension, was divided into four wells of a qPCR plate, with 50 μl in each well, and 2.5 μl of 20× EvaGreen (Biotium) per well was added. Lasy, qPCR was performed until reaching the nonexponential plateau phase.

The magnetic beads were preserved for potential future use. After the template switch reaction, the beads were resuspended in 250 μl of tris-T buffer and carefully stored at 4°C. This storage condition ensured the stability and integrity of the beads for potential subsequent applications.

#### Day 4: Tagmentation and round 4 of barcoding

The qPCR reactions were subjected to solid phase reversible immobilization (SPRI) size selection using Kapa Pure Beads (Roche) at ratios of 0.8× and 0.7×. This step effectively removed fragments smaller than 300 bp. The concentration of the resulting libraries was determined using a Qubit fluorometer (Thermo Fisher Scientific), and the fragment distribution was assessed using an Agilent 2100 Bioanalyzer with the Agilent High Sensitivity DNA Kit.

Next, the sublibraries underwent tagmentation using the Nextera DNA Library Preparation Kit (Illumina). The tagmentation process was promptly neutralized using the Monarch PCR & DNA Cleanup Kit (NEB). The samples were then eluted in a final volume of 20 μl using the elution buffer.

For the fourth and final round of barcoding and library preparation, PCR amplification was performed using tagmentation master primer i7 and library-specific tagmentation primers i5. The resulting products were subjected to size selection using Kapa Pure Beads (Roche) at ratios of 0.7× and 0.6×. The fragment distribution was once again assessed using the Agilent 2100 Bioanalyzer with the Agilent High Sensitivity DNA Kit, and the library concentrations were quantified using the Qubit fluorometer.

### SPLiT-seq read processing

Following sequencing on a NovaSeq 6000 platform (Illumina) by Novogene, our generated data underwent quality control and preprocessing steps to ensure high-quality data for subsequent analyses. These steps included read alignment, barcode demultiplexing, and removal of low-quality or duplicate reads. The sequencing was performed with 150–base pair length, paired-end reads. However, the reads were provided without any extensive quality verification apart from a basic quality check. Therefore, we conducted initial quality checks using the FastQC software. To further improve the quality of the data, we utilized the CutAdapt v2.1 tool. For read 1, we removed Illumina universal adaptors, as well as short and low-quality reads, using the command: “cutadapt -b AGATCGGAAGAG -m 60 -j 4.” Similarly, for read 2, we removed short and low-quality reads, terminal Ns, and the Nextera adapter sequence using the command: “cutadapt -m 94 -j 4 --trim-n -b CTGTCTCTTATA.” To ensure that only paired reads were retained, we used the Makepairs tool in our preprocessing pipeline. This step helped us maintain the integrity of paired-end reads for subsequent analyses.

We used a new *S. mediterranea* genome and annotation by the J. Rink laboratory (*52*). After the preprocessing steps, we utilized the Drop-seq_tools-2.3.0 software package (available at https://github.com/broadinstitute/Drop-seq) to create sequence dictionary, refFlat, reduced GTF, and interval files. To generate the STAR-2.7.3a index (*73*), we used the following settings: --sjdbOverhang 99, −-genomeSAindexNbases 13, and --genomeChrBinNbits 14. This index allows for efficient mapping of the reads to the reference genome. Each of the two sublibraries sequenced in our study was processed separately. For barcode extraction, verification, and correction (with hamming distance ≤ 1), we used the SPLiTseq toolbox (available at https://github.com/RebekkaWegmann/splitseq_toolbox). This toolbox incorporates many of the components of Drop-seq_tools-2.3.0. Mapping of the processed reads was performed using STAR-2.7.3a with the --quantMode GeneCounts option and default settings. To re-order and merge the aligned and tagged reads, we used Picard v2.21.1-SNAPSHOT (developed by the Broad Institute, available at http://broadinstitute.github.io/picard/). Specifically, we used the SortSam and MergeBamAlignment tools. For further annotation of the mapped reads, we used the Drop-seq_tools-2.3.0 TagReadWithInterval and TagReadWithGeneFunction tools in sequential order. These tools use the custom refFlat and genes.intervals files created earlier to note the mapping location and assign gene function to the reads. The resulting mapping files were then used to create expression matrices for each library individually using the Drop-seq_tools-2.3.0 DigitalExpression tool. We applied the following settings: READ_MQ = 0, EDIT_DISTANCE = 1, MIN_NUM_GENES_PER_CELL = 50, and LOCUS_FUNCTION_LIST = INTRONIC. These settings ensured the generation of accurate expression matrices for subsequent analyses.

### Genome annotation

The *S. mediterranea* genome (*52*) and annotation were subjected to a snakemake workflow of standard tools for gene annotation, found at https://github.com/apposada/gene_annot. Briefly, the longest isoforms per gene were extracted using gffread on an AGAT-standardized version of the GFF3 annotation file and the genome sequence FASTA. These sequences were translated into proteins using TransDecoder (https://github.com/TransDecoder/TransDecoder) using evidence from BLAST reciprocal best hits against the UniProt database and hmmer queried against the PFAM database, as previously described (*51*). Protein sequences were further annotated using (i) eggNOG against the protein set of all metazoa (*74*); (ii) InterProScan against the PFAM, SFAM, and Panther databases; (iii) BLAST reciprocal best hits against the UniProt database; and (iv) assigning co-orthologs from a set of model organism species using OrthoFinder (*75*). Evidence from (ii, iii, and iv) was used to annotate transcription factors into TF classes using also data from AnimalTFDB 3.0 (*76*), as previously described (*51*).

### Single-cell transcriptomic analysis

The scRNA-seq data were analyzed using Scanpy. The gene expression data from each of the two sublibraries, encapsulated in the 10x matrices, were uploaded and processed to associate the sample names with the Anndata object. We tagged each cell observation in adata.obs with its sample of origin. In addition, each gene in the adata.var. was tagged with the previously described gene annotations.

We applied additional quality control (QC) measures to the scRNA-seq data to eliminate low-quality cells. Specifically, we used the command “sc.pp.filter_cells(adata, min_counts = 100)” to filter out cells with a low number of UMIs and “sc.pp.filter_cells(adata, min_genes = 100)” to filter out cells with a low expression of genes. To further assess the quality of the data, QC metrics were calculated using the command “sc.pp.calculate_qc_metrics(adata, qc_vars=[‘mt’], percent_top=None, log1p=False, inplace=True).” Cells with a low number of expressed genes are often indicative of poor quality or technical artifacts, and therefore, they were excluded from further analysis. Similarly, cells with a low total UMI count may be associated with low RNA content or technical issues, so they were also excluded to improve the downstream analysis quality. To achieve this, we filtered the matrix in the Anndata object, removing cells where the number of expressed genes was less than 900 using the command “adata = adata[adata.obs.n_genes_by_counts < 900,: ]” and cells where the total molecule count was less than 1200 using the command “adata = adata[adata.obs.total_counts < 1200,: ].” Furthermore, we performed normalization to ensure consistent read counts across cells, which was accomplished using the command “sc.pp.normalize_total(adata).” Subsequently, the data were log-transformed to stabilize the variances, and variable genes were identified to pinpoint genes that exhibited significant expression variation across cells. For this purpose, the top 24,000 variable genes were selected using the command “sc.pp.highly_variable_genes(adata, n_top_genes = 24000).”

To reduce the dimensionality of the dataset, we used PCA and UMAP (for dimension reduction). PCA was performed on the first 150 principal components using the command “sc.tl.pca(adata, svd_solver=‘arpack’, n_comps=150).” An elbow plot was created to assess the amount of variance captured by these components. Based on the PCA results, the neighborhood relationships between cells were determined using the first 95 principal components with the command “sc.pp.neighbors(adata, n_neighbors=55, n_pcs=95).” For UMAP visualization, the calculated neighbors were used, and parameters such as minimum distance and spread were specified to control the density and layout of the visualization. This was accomplished using the command “sc.tl.umap(adata, min_dist=0.75, spread=1.25, alpha=1, gamma=1.0).” Clustering was performed using the Leiden algorithm, and the resolution was set to values 1, 2, 3, and 4 to determine the optimal granularity of the clusters. To evaluate the consistency and robustness of the clustering results, correlation matrices and dendrograms were examined for each clustering resolution. Reliable clustering was indicated by consistent and well-separated clusters in both the correlation matrix and dendrogram. In addition, known marker genes were referenced to assess whether the clustering algorithm accurately captured the expected biology (*44*). Last, to identify marker genes associated with each cluster, gene rankings were generated using both the ‘wilcoxon’ and ‘logreg’ methods. The cluster dendrogram was obtained with the scanpy function “sc.pl.dendrogram.”

To gain further insights into the cellular composition and heterogeneity present in the scRNA-seq data, we visualized cluster-specific marker genes using dot plots, heatmaps, and features plots. These visualizations provided a concise representation of gene expression within each cluster as well as across clusters. By performing these analyses and examining the expression patterns of known marker genes, we were able to identify distinct cell populations and infer their respective cell types. To obtain log_2_ ratios between cell numbers in L and S planarians, we divided the number of cells in each cluster in each size category by the sum of the cells in the category. We then applied logarithms and obtained the ratios by subtracting the log frequency values. These values were plotted on the UMAP using a custom color scale, since most values are between −1.3 and 1.1 except cluster 54 (secretory 4), which has an outlier value of 4.4.

### Pseudo-bulk and differential gene expression analysis

Using the cell annotation derived from our single-cell analysis (i.e., clustering, broad cell types, etc.) as well as cell information such as size of the organism of origin (L, M, and S) and cell library, we generated a pseudo-bulk matrix by aggregating, for a given gene X, all the counts of gene X from cells coming from the same cluster and library, effectively generating a pseudo-bulk matrix with genes in rows and “pseudosamples” (i.e., all combinations of cell type + “condition,” i.e., organism size + “replicate,” i.e., library) in columns. For a given cell type i, we subsetted this pseudo-bulk matrix to keep pseudosamples from cell type i and genes annotated as “high confidence” in the gene annotation (see Materials and Methods) and performed differential gene expression analysis using DESeq2 (*66*) inside a custom wrapper. Briefly, we filtered genes with less than two counts in at least one replicate and performed a negative binomial test from DESeq2 with contrast “L” over “S.” Genes were considered differentially expressed if the *P* value of the test was lower than 0.05.

### In situ hybridization images

All in situ hybridization images were downloaded from https://digiworm.wi.mit.edu/ (*42*). The genes selected are dd_Smed_v4_21541_0_1 (h1SMcG0010053), dd_Smed_v4_750_0_1 (h1SMcG0017679), dd_Smed_v4_43_0_1, (h1SMcG0005326), and dd_Smed_v4_7593_0_1 (h1SMcG0011854).

### Graph neighborhood differential abundance

Comparison in cell-state abundance between the different sizes of the organism of origin were analyzed using the package Milo (*54*) with the implementation in python milopy (https://github.com/emdann/milopy). The dataset was subsetted into cells from each corresponding library and grouped into adata objects for the two differential comparisons made (L versus S and M versus S). kNN graphs were constructed using similarity of each cell embedding, and cells were classified into neighborhoods. Later, this information was assigned to the cluster annotation of the whole dataset based on the number of cells belonging to that neighborhood. To detect significance across the pairs in the differential neirghbohood analysis a threshold of spatial false discovery rate (<1%) and log fold change (±0.5) was implemented. For more details in the commands used for these analyses, see the code repository.

### Statistical analysis

To assess the relationship between cluster memberships and size category membership, Pearson’s chi-squared tests for independence were conducted. Each omnibus chi-squared test was followed by a post hoc analysis of the standardized residuals to assess which category combinations were significantly over- or under-represented. Multiple testing was corrected using the Benjamini-Hochberg correction, which was deemed more appropriate than the more commonly used Bonferroni correction as it focused on reducing false discovery rate under multiple testing.

All statistical analysis was conducted in R version 4.3.0 (R Core Team, 2023; www.R-project.org/) using RStudio 2023.03.1 + 446 (Posit team, 2023; www.posit.co/). Additionally to baseR functionalities, the package suite tidyverse version 2.0.0 (*77*) and packages chisq.posthoc.test version 0.1.2 (Ebbert, 2019; https://CRAN.R-project.org/package=chisq.posthoc.test), colorspace version 2.1-0 (*78*), and rstatix version 0.7.2 (Kassambara, 2023; https://CRAN.R-project.org/package=rstatix) were used for analyses and development of heatmaps.

### Comparison with anterior-posterior dataset

To compare our observations with those of the anterior-posterior RNA-seq dataset performed by Stückemann and colleagues (*65*), we first obtained the best markers of each of our resolution 3 Leiden clusters. These were defined by the overlap between the Wilcoxon and the logistic regression top 30 markers. These markers were parsed using our annotation of the *S. mediterranea* genome (see project repository) using pandas scripts to obtain their equivalent transcripts in the Dresden version 6 transcriptome. Then, the values of these transcripts in the anterior-posterior dataset were obtained, *z*-scored, and averaged. The results were displayed as a heatmap using pandas and seaborn. The reverse comparison was performed by parsing the Dresden v6 transcripts against the newer version of the genome using pandas. Then, we used scanpy sc.tl.score_genes to obtain scores for each of the Stückemann *et al.* (*65*) in our cells. These scores were visualized in a matrixplot using scanpy.

### Real-time qPCR

L, M, and S planarians were classified as described above. Three biological replicas for each condition were used. Total RNA was extracted from each sample using TRIzol reagent (Invitrogen), following the manufacturer’s protocol. cDNA was synthesized from 1 mg of RNA using the Maxima H Minus Reverse Transcriptase (Thermo Fisher Scientific) and anchor oligo(dT)20 V. The samples were incubated for 35 min at 50°C, diluted 1:10, and kept at −20°C until use. The top and most specific markers of selected clusters were chosen for the qRT-PCR. The housekeeping gene (cytoplasmic dynein 1 heavy chain, DYNC1H1) was picked from the single cell dataset by extracting the most expressed gene, with the lowest log fold change expression between L, M, and S samples. Primers were designed using Primer3 software, and sequences can be found in data S8. The qRT-PCR reaction was performed using the Luna Universal qPCR Master Mix (New England Biolabs) in a QuantStudio 1 Real-Time PCR System (Thermo Fisher Scientific). For each biological replicate, we inputted three technical replicates in every reaction. Log_2_^-∆∆*C*T^ values and plots were obtained using Python.
