# Supplementary material for: Allometry of cell types in planarians by single-cell transcriptomics
Source: Sci Adv. 2025 May 7;11(19):eadm7042. doi: 10.1126/sciadv.adm7042 (PMC12057665; doi:10.1126/sciadv.adm7042)
Supplement: Supplementary file 1 — Supplementary Text Figs. S1 to S9 Legends for data S1 to S9 [file sciadv.adm7042_sm.pdf]

Science Advances
AAAS

Supplementary Materials for

Allometry of cell types in planarians by single-cell transcriptomics

Elena Emili *et al.*

Corresponding author: Jordi Solana, j.solana@exeter.ac.uk

Sci. Adv. **11**, eadm7042 (2025)
DOI: 10.1126/sciadv.adm7042

The PDF file includes:

Supplementary Text
Figs. S1 to S9
Legends for data S1 to S9

Other Supplementary Material for this manuscript includes the following:

Data S1 to S9

Supplementary Text

Supplementary Note: **Power Analysis of cell abundances in SPLiT-Seq data**

Introduction

In statistics, power is a measure to assess the likelihood of effectively rejecting the null hypothesis provided the circumstances in which the test was done. The power of a test is related to the sample size, the criterion of significance of choice, the degrees of freedom of the test, and the so called "effect size" (ES, a number to estimate how present in the population is the biological phenomenon of interest). Generally, small sample sizes require larger effect sizes for a test to be considered powerful, but this may not hold true for very large samples (such as a whole dataset of planarians in a single cell experiment, as it is our case).

There are many ways to estimate the ES, generally more than one per kind of test. For proportion comparisons such as the Chi-Square post-hoc analyses we perform in Figure 4, a standard ES is an index that scales in relation to the discrepancies between the observed proportions of a sample and the expected proportions of a population, in an R x K contingency table of categories.

A priori power analysis of a putative dataset

The body of a complex, multicellular organism such as an animal is formed by many different cell types, but this does not mean these cell types are equally abundant or frequent. We know from multiple single-cell transcriptomic studies that several cell types, especially those like secretory cells, sensory cells, or several neuronal and intestinal subtypes, are less abundant than those such as epidermis, skeletal muscle or cholinergic neurons, in a disproportionate order of magnitude (*42*, *44*). In practice this means that, if we were to study a phenomenon that entails changes in the relative abundances of cell types, some cell types will see more subtle differences in abundances than others. Because no sequencing technology can capture a perfect molecular portrayal of a biological sample, this means that our capacity to detect such subtle differences may be hindered by factors like sequencing depth, or the amount of biological sample needed to capture those smaller cell types at a given sequencing depth. Therefore, it becomes necessary to assess to which extent one can rely on abundance tests to detect such differences under a given experimental design, because there might be not a single rule-of-thumb. For this we can calculate the *a priori* power of the abundance tests performed in such clusters.

Let us consider a putative single cell dataset of *Schmidtea mediterranea*, comprised of 25,000 cells from four different samples, each being a different experimental condition, and equally abundant –each comprising 25% of the total dataset (Supplementary Figure S5A). Planarian cell type abundance varies a lot: it ranges from small clusters that comprise below one percent of the total dataset (secretory cells, several neuronal cell types) to large clusters sitting comfortably around or above the ten percent (muscle cells, neoblasts, cholinergic neurons).

Of these four conditions, let us suppose one condition, named 'X', has different cell type abundances than what one might expect based on the literature. If we tested for differences in abundances of a small cluster 'Y' (which normally comprises 0.05% of the dataset) between

different conditions, we could think of a contingency table like the one in Supplementary Figure S5B.

Of note that the marginal proportions for belonging to the small cluster are not 0.5% and 99.5%, as one might expect from the literature. This is because this dataset is comprised of several samples, one of which (X, the one we are testing) has a deviation from the expected proportions which alters the relative abundance of the cell type in this specific dataset (the other three still following the expected cell proportions in this example). Likewise, we assume 25% and 75% for the frequencies of cells from (or not from) condition X as we mentioned we have an equally large number of cells from each condition.

In this putative situation, we could find that cells from condition X have 0.6% cells Y, compared to cells from the rest of the conditions which retain an expected value of 0.5% in the dataset. This would mean that we found differences of 0.1% between our condition of interest and the expected proportions (Supplementary Figure S5B). Are these differences enough to reliably reject the null hypothesis if we saw ourselves in such a case?

In order to assess the power of this test, we must calculate the following:

1. Sample size: As this is a Chi-Square comparing differences in small cluster 'Y' relative to the whole dataset, the sample size would correspond to the total amount of cells (25000).
2. Significance criterion: all the tests reported as significant in our study have a p-value below 0.05.
3. Degrees of freedom: this test consisted of two categories: "being Y / not being Y" (category R) and "condition X / not condition X" (category K). The resulting contingency table is 2x2 and thus the degrees of freedom are (2-1) x (2-1) = 1 x 1 = 1.
4. The Effect Size. We relied on Cohen's formula for calculating the effect size 'w' (*55*), for which we need to retrieve the observed proportions (which we already have) and the expected proportions. See below for more details.

To retrieve the expected proportions, we can think of the following. If the null hypothesis (expected) was true, there would be **no association** between being from cluster Y and being from condition X. Therefore, if there were no association, the **individual proportions would be expected**. In other words: 25% of the cells in the dataset are from condition X, and .525% of cells in the dataset are from the cluster Y. If there were no association, we expect to find the same fraction of cells Y inside the fraction of cells from condition X. Which means, the expected probability of cells Y in planarians from condition X would be .525% of the 25% of cells of the population. In practice, this means we retrieve the expected proportions by multiplying the marginals of the contingency table of proportions P1 (i.e. the table of observed proportions). This is the P0 table, the table of expected proportions (Supplementary Figure S5C).

As stated before, the "Effect Size" is a way to quantify, or parametrise, the presence of the phenomenon in the population. This stems from the idea that oftentimes a null hypothesis is tethered to a specific quantitative aspect of the population: for example, the **null** hypothesis of differences being prone to a given disease between biologically-assigned-at-birth males and females might be .50, i.e. no differences. Following this logic, any quantitative deviation from this proportion can be understood as a means to parametrise the degree to which the null hypothesis is false. According to Cohen (*55*), when the null hypothesis is incorrect, the truth deviates from the null hypothesis by a specific amount. This degree of deviation reflects how

strongly the phenomenon being studied is present. The effect size (ES) is introduced as a way to quantify this. When the null hypothesis is accurate, the effect size is zero, but when it is false, the effect size takes on a nonzero value that represents the extent of departure from the null hypothesis. Essentially, the effect size serves as a measure of how much the observed data diverges from what would be expected under the null hypothesis.

In the case of testing the association between being “cell type Y” and belonging to “condition X”, we could think of the ES as the departure of the observed proportions from the expected proportions. Cohen implemented a formula to calculate this:

Equation 1:

$$\sum_{i=1}^{m} \sqrt{\frac{(P_{1i} - P_{0i})^2}{P_{0i}}} = w$$

Where ‘P_{1i}’ corresponds to observed proportions and ‘P_{0i}’ corresponds to expected proportions, and the range from 'i' to 'm’ are the different cells from the contingency tables of observed and expected proportions. That is, the different values of the contingency table, which correspond to combinations of categories from R and K.

Considering a simplified depiction of the two contingency tables P1 and P0, where every cell is labelled with one letter, and colours indicate whether it is P1 (observed) or P0 (expected). Our visual depiction in Supplementary Figure S5D can help us visualise the way Cohen’s ‘w’ parameter is calculated.

With this, we can therefore estimate that, in a dataset of 25,000 cells and four evenly sampled experimental conditions, observing a small cluster going from 0.5% to 0.6% translates into an effect size of ~0.006. In turn, using the `pwr.chisq.test` function from the R package `pwr`, providing the rest of required values (sample size, significance level, degrees of freedom) we can estimate this translates into a statistical power of .15, which is not considered enough.

We can expand this reasoning to more scenarios, with multiple differences in proportions across clusters of different size (abundance in the dataset), in order to explore which differences would yield powerful tests for each kind of cluster. With these values we could guide ourselves if we found similar differences in abundance. The resulting effect size and power analyses can be observed in the heatmaps of Supplementary Figure S5E.

On the left side we have a heatmap showing the estimated Effect Size for clusters of different sizes (rows) when detecting putative differences of different orders (columns). On the right side we have a heatmap for the resulting power analysis of the respective Chi-Squared tests. One asterisk (”*”) indicate power above 0.8, and two asterisks (”**”) indicate power above 0.95. As a general rule of thumb, any difference above two percent (that is, finding an increase or a decrease of 2%) can reliably yield a powerful test, below which these differences vary with the cluster size. The smaller the cluster, the more subtle these differences and the less likely they could be attributed to variability in the dataset that is not related to the phenomenon under study.

A posteriori **power analysis: case example from our dataset**

With this knowledge, how does our resulting Chi-Squared tests of abundances relate to these putative scenarios?

In our case, provided the number of tests and comparisons are made, we will assess the power of every test in the heatmaps of Figure 4. But for clarity's sake, we will focus on just one of these tests.

A brief clarification before continuing. We will be referring to the major groups of cell types as "**broad types**" –these are: **neoblasts, epidermal, phagocytes, basal/goblet cells, muscle, neurons, parenchyma, protonephridia,** and **secretory** cell types. By opposition, we will refer to the discrete clusters used throughout the manuscript as "**specific cell types**" –these are neoblasts 1, neoblasts 2, committed neoblasts, germ line progenitors, early epidermal progenitors, etc.

As one example, we will focus in one such test. In Figure 4C, we can see that the "specific cell type" neoblasts 2 is significantly enriched in small planarians and depleted in large planarians. To assess the power of this test, we must figure out the following data:

1. Sample size: as before, the sample size would correspond to the total amount of cells (28423).
2. Significance criterion: again, this would be 0.05.
3. Degrees of freedom: again, this would be 1.
4. The Effect Size, which we can estimate again using Cohen's formula.

More details on the effect size. Suppose the following contingency table, where we study the number of "neoblasts 2" cells in small planarians. We have two different ways to categorise the cells: first, the cell type they belong to; second, the size of the planaria they come from. The first category we call R (with categories r1 and r2), and the second category we call K (with categories k1 and k2) (Supplementary Figure S5F). In this table of number of cells, "not small" amounts to cells from M and L planarians, and "not neoblasts" amounts to cells from every other specific cell type except neoblasts2.

To retrieve the proportions within the sample, we can divide these numbers by the total, 28423. This is what we call the P1 proportions table (Supplementary Figure S5G).

Like before, we retrieve the expected proportions by multiplying the marginals of the contingency table of proportions P1. This leads to the expected proportions P0 (Supplementary Figure S5H).

We will apply Cohen's formula here, replacing the different values:

Equation 2:

$$\sqrt{\frac{(.0177-.0149)^2}{.0149}+\frac{(.0371-.0399)^2}{.0399}+\frac{(.2538-.2566)^2}{.2566}+\frac{(.6914-.6886)^2}{.6886}}=.0276$$

According to Cohen's tables of effect size values, as a rule of thumb, low ES values are .1, medium ES values are .3, and high ES values are .5. Following this criterion, the value obtained above should be understood, in principle, as negligibly low. However, we must remember that a power analysis also takes in other variables such as the sample size, and the significance criterion.

When plugging in these values into the R function `pwr.chisq.test` from the R package `pwr`, we retrieve the results observed in Supplementary Figure S5I. We see that this large sample size, despite a very low ES value, results in very high power (a standard value of 'good' power analyses is considered equal or above 0.8). This means that in our dataset, with this

experimental design, we can reliably reject the null hypothesis that there is no relationship between being a cell from the “neoblasts 2” specific cell type and coming from small planarians.

A posteriori analysis of differences in abundances for other cell types

This also happens with the rest of statistically significant abundance tests performed for other specific cell types. Supplementary Figure S5J represents the resulting power analysis of every Chi-Squared test in Figure 4C. Dashed red line indicates power = 0.8. We also retrieve good power when doing the same for all the significant tests at the broad cell type level (Supplementary Figure S5K), and the same for significant tests when comparing the abundances of specific cell types relative to their broad type, with one exception (phagocyte progenitors 1 in L, which falls right below 0.8)(Supplementary Figure S5L).

With this, we focused on trying to assess if there was any association between cluster size and having relatively good power in our dataset. For this we visualised changes in cluster size (logarithmic) against the resulting power of each of the tests Figure 4C (Supplementary Figure S5M). There seems to be a small relationship between cluster size and level of power ($R = +.28$, highlighted by the blue line).

Conclusion

Our *a priori* analysis suggested that differences in abundances can be reliably detected even for small clusters, for which smaller differences may tend to be significant. This aligns with the high power detected *a posteriori* for all the significant tests of abundances performed in this manuscript. There is room for improvement, however: the slight linear relationship between cluster size and statistical power aligns with the idea that detecting differences is influenced by the resolution of the data (depth of sequencing, for example). This means that, with more material and deeper sequencing, we can likely enhance the detection of smaller clusters and increase our capability to assess differences in cell abundance across experimental conditions.

With this, overall, we think the large sample size of a single cell experiment suffices to detect subtle changes in cell abundances of different clusters, regardless of the cluster sizes we normally observe in planarians.

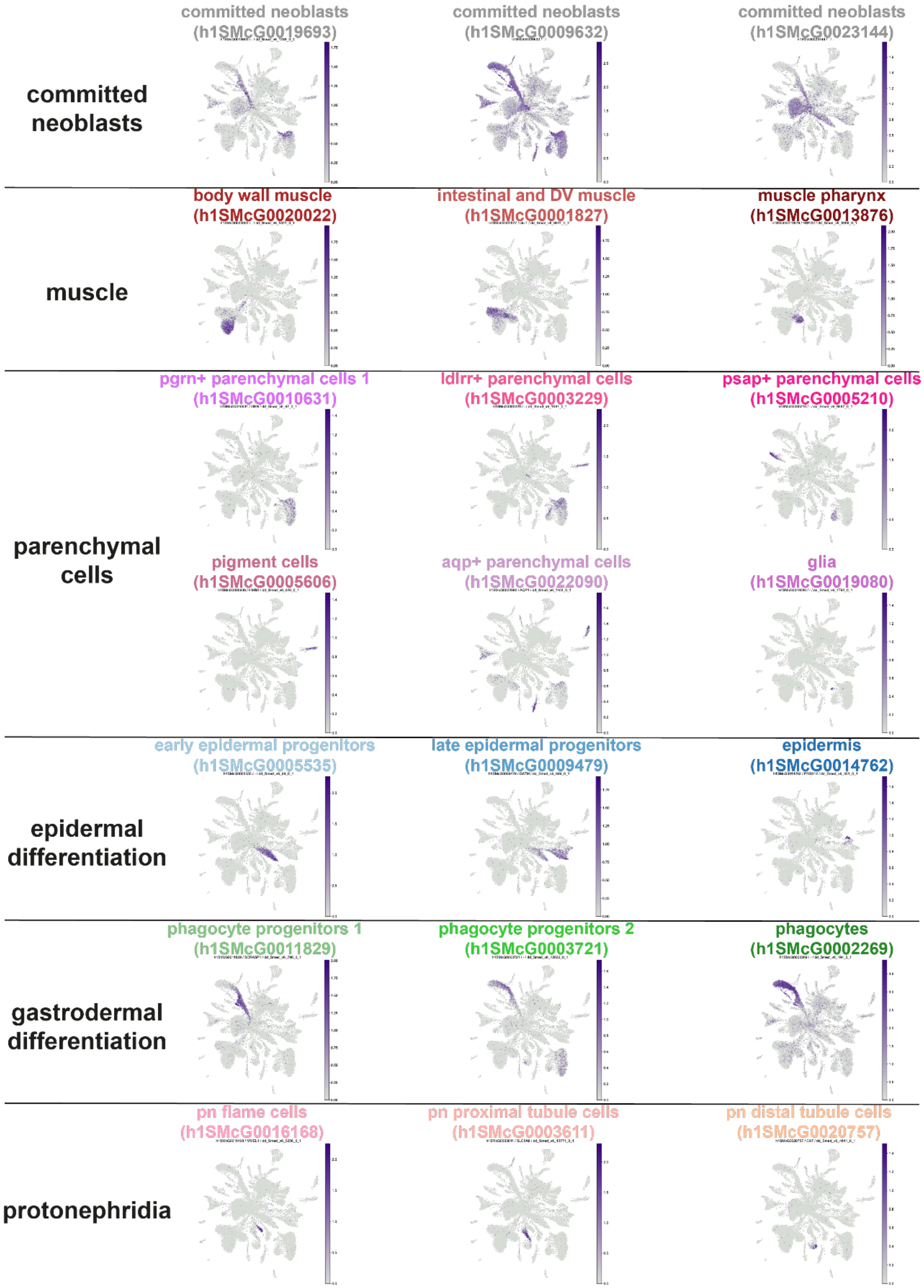

Fig. S1.

Cluster Annotation. UMAP feature plots of markers of specific cell clusters used to annotate different cell types..

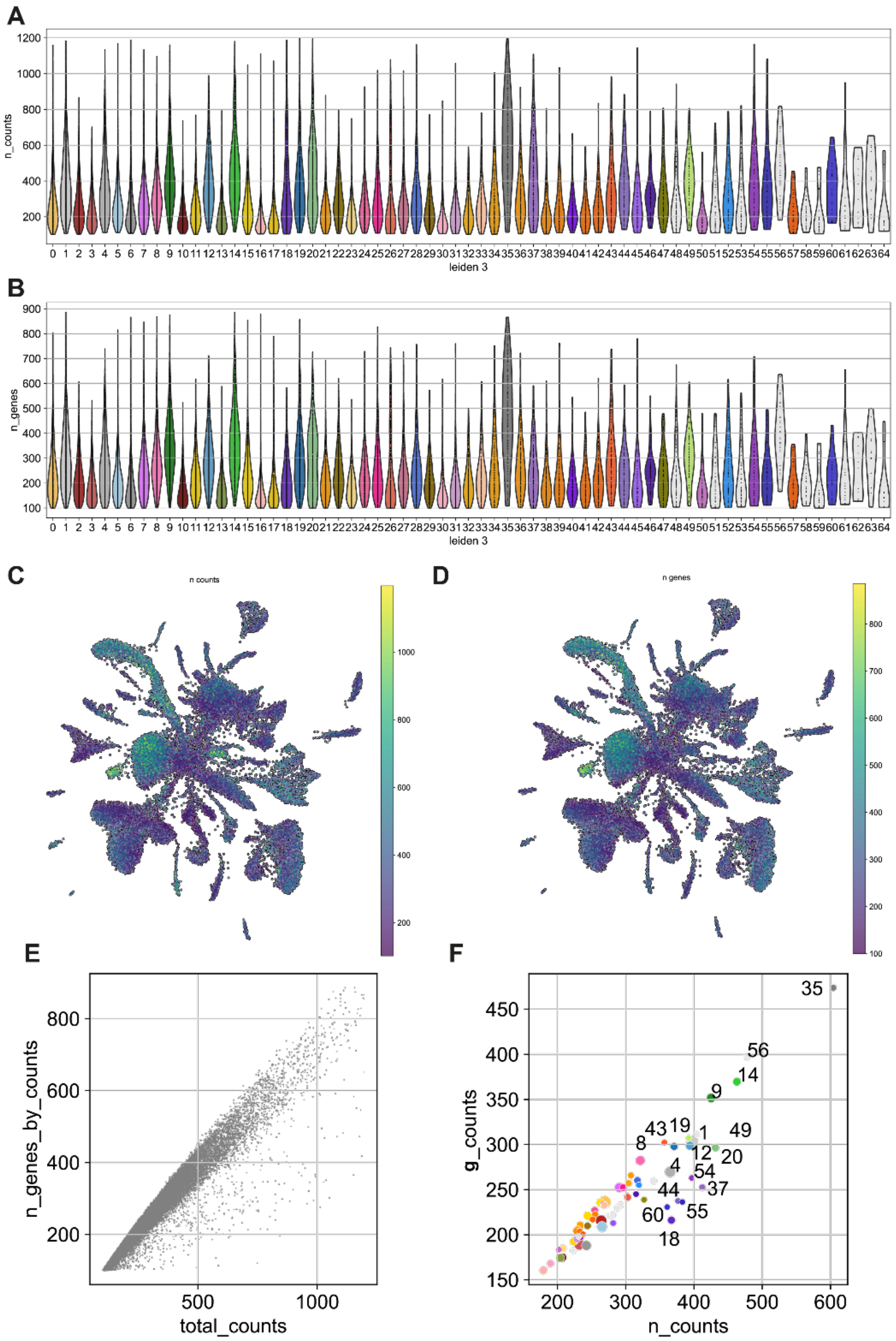

Fig. S2.

Sizes dataset metrics. A: Violin plots showing the number of UMI counts detected per cell in each cluster. B: Violin plots showing the number of genes detected per cell in each cluster. C: UMAP showing the distribution of UMI counts detected per cell. D: UMAP showing the distribution of genes detected per cell. E: Scatter plot of total number of counts vs. number of genes per cell. F: Scatter plot of average of UMI counts per cluster vs. average of genes per cluster.

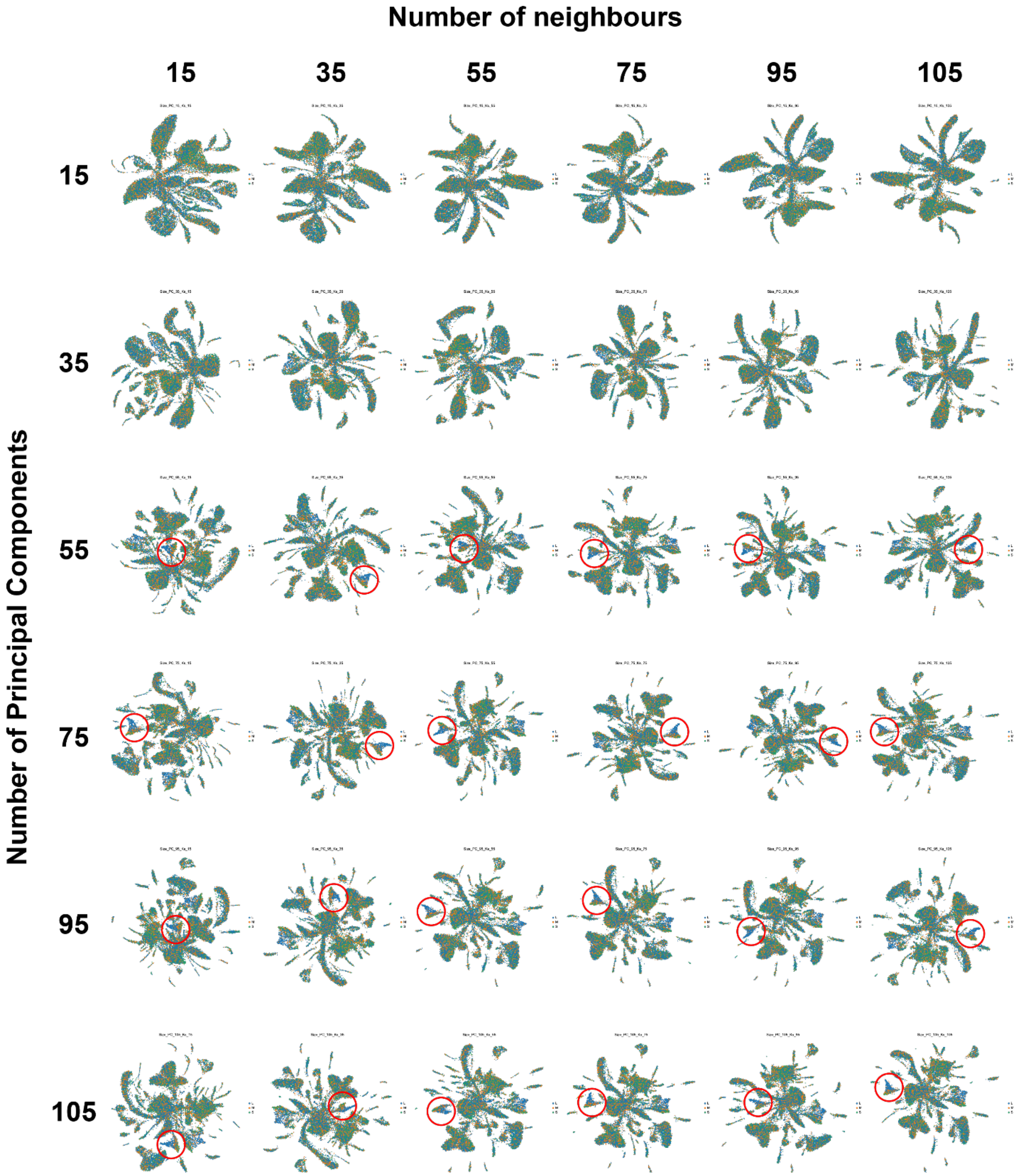

Fig. S3.

Pre-processing parameter space exploration. UMAP visualisation of the 28,738 cells pre-processed with different parameters for number of neighbours (15-105), and number of principal components (15-105). Red circle indicates the basal cells.

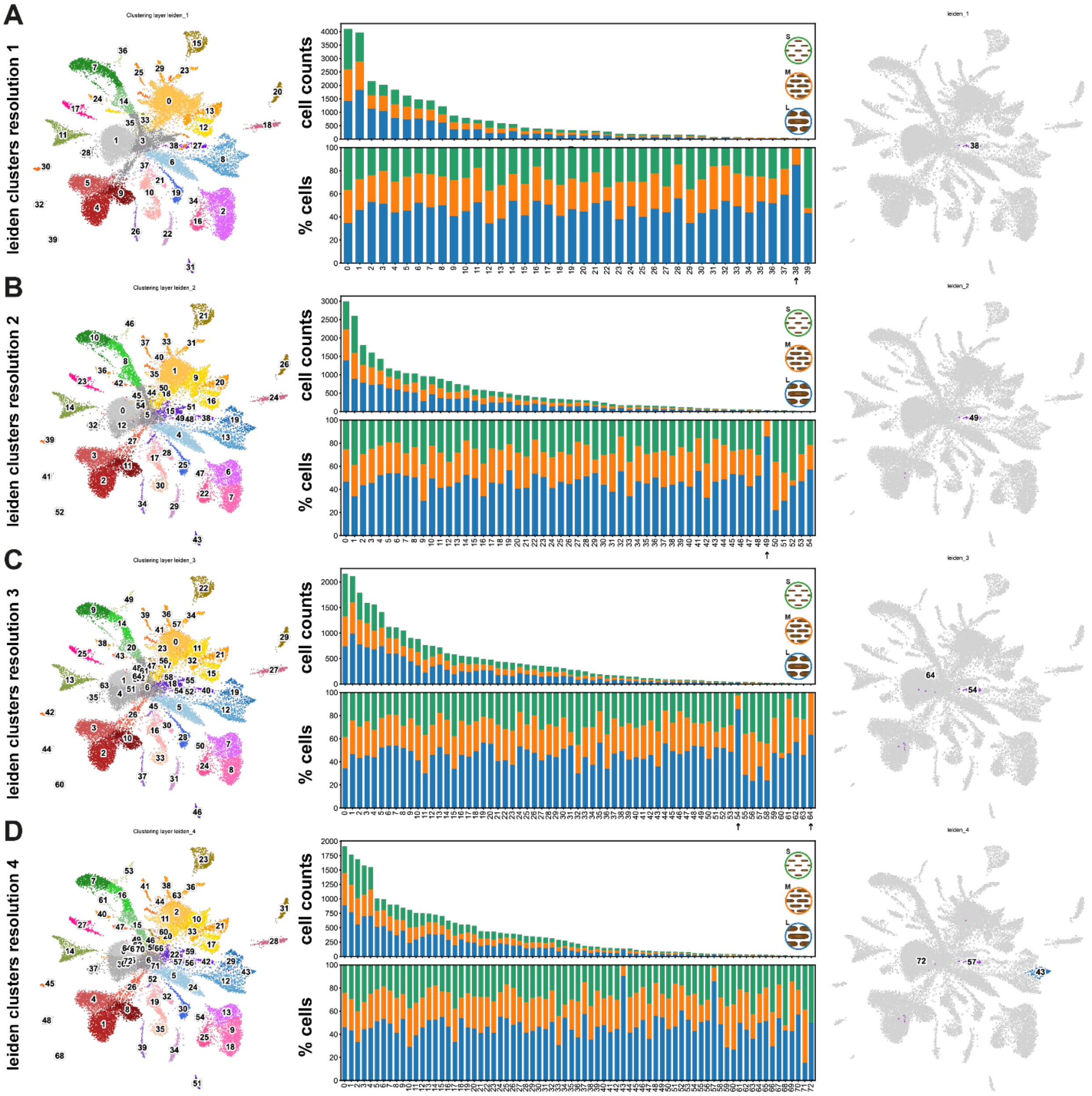

Fig. S4.

Cell type frequency in planarians of different sizes. A-D: UMAP, stacked barplot of cell counts and cell percentages based on Leiden clustering at resolution 1 (A), 2 (B) 3 (C) and 4 (D). Arrows point to the clusters with the lowest percentages. UMAPs highlighting cell clusters with the lowest percentages are depicted in the right.

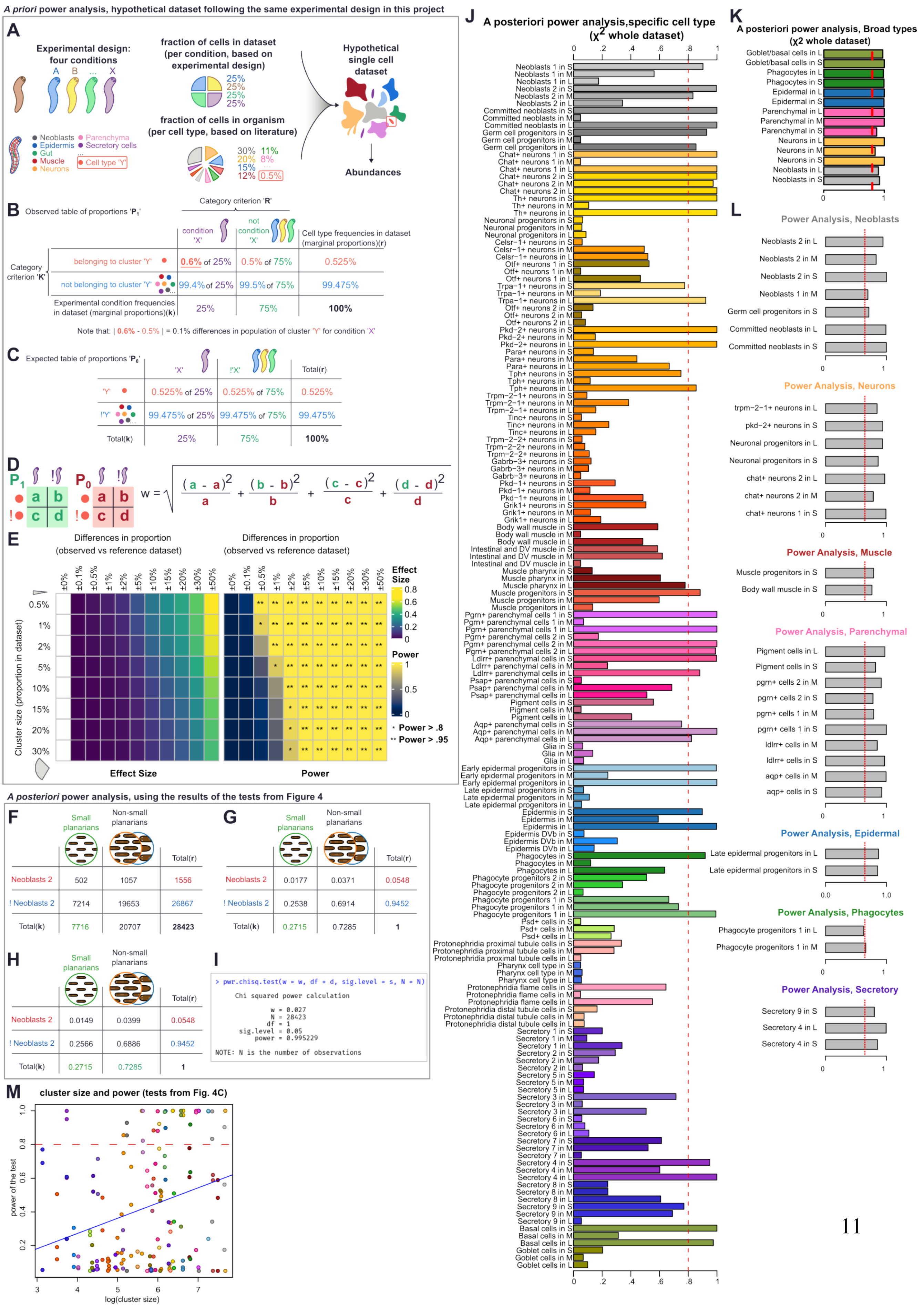
A priori power analysis, hypothetical dataset following the same experimental design in this project
A
Experimental design: four conditions
A B ... X
fraction of cells in dataset (per condition, based on experimental design)
25% 25% 25% 25%
fraction of cells in organism (per cell type, based on literature)
30% 11% 20% 8% 15% 12% 0.5%
Hypothetical single cell dataset
Abundances
Neoblasts Parenchyma Epidermis Secretory cells Gut Muscle Cell type 'Y' Neurons
B Observed table of proportions 'P1'
Category criterion 'R'
condition 'X'
not condition 'X'
Cell type frequencies in dataset (marginal proportions)(r)
Category criterion 'K'
belonging to cluster 'Y' 0.6% of 25% 0.5% of 75% 0.525%
not belonging to cluster 'Y' 99.4% of 25% 99.5% of 75% 99.475%
Experimental condition frequencies in dataset (marginal proportions)(k) 25% 75% 100%
Note that: | 0.6% - 0.5% | = 0.1% differences in population of cluster 'Y' for condition 'X'
C Expected table of proportions 'P0'
'X' !'X' Total(r)
'Y' 0.525% of 25% 0.525% of 75% 0.525%
!'Y' 99.475% of 25% 99.475% of 75% 99.475%
Total(k) 25% 75% 100%
D
P1 a b c d
P0 a b c d
w = √((a - a)²/a + (b - b)²/b + (c - c)²/c + (d - d)²/d)
E
Differences in proportion (observed vs reference dataset)
±0% ±0.1% ±0.5% ±1% ±2% ±5% ±10% ±15% ±20% ±30% ±50%
Cluster size (proportion in dataset)
0.5% 1% 2% 5% 10% 15% 20% 30%
Effect Size
Power
* Power > .8
** Power > .95
A posteriori power analysis, using the results of the tests from Figure 4
F
Small planarians / Non-small planarians / Total(r)
Neoblasts 2 502 1057 1556
! Neoblasts 2 7214 19653 26867
Total(k) 7716 20707 28423
G
Small planarians / Non-small planarians / Total(r)
Neoblasts 2 0.0177 0.0371 0.0548
! Neoblasts 2 0.2538 0.6914 0.9452
Total(k) 0.2715 0.7285 1
H
Small planarians / Non-small planarians / Total(r)
Neoblasts 2 0.0149 0.0399 0.0548
! Neoblasts 2 0.2566 0.6886 0.9452
Total(k) 0.2715 0.7285 1
I
> pwr.chisq.test(w = w, df = d, sig.level = s, N = N)
Chi squared power calculation
w = 0.027
N = 28423
df = 1
sig.level = 0.05
power = 0.995229
NOTE: N is the number of observations
M cluster size and power (tests from Fig. 4C)
power of the test
log(cluster size)
J A posteriori power analysis,specific cell type (χ2 whole dataset)
K A posteriori power analysis, Broad types (χ2 whole dataset)
L
Power Analysis, Neoblasts
Power Analysis, Neurons
Power Analysis, Muscle
Power Analysis, Parenchymal
Power Analysis, Epidermal
Power Analysis, Phagocytes
Power Analysis, Secretory

Fig. S5.

Statistical power analysis in single cell transcriptomics data of *S. mediterranea*. A: Design of a putative single cell dataset for an *A priori* power analysis. Like our dataset, we suppose conditions sampled evenly, and expected cell type proportions following reference literature. We focus on a putative cell cluster ‘Y’ changing abundances in condition ‘X’. B: Contingency table of hypothetical observed proportions ‘P1’ for cluster ‘Y’ in condition ‘X’. C: Contingency table of hypothetical expected proportions ‘P0’ for cluster ‘Y’ in condition ‘X’. D: Visual depiction of how the effect size is calculated. Green contingency table represents observed proportions (P1); red contingency table represents expected proportions (P0). E: Heatmaps showing putative effect sizes and powers of chi-squared tests for a range of abundance differences and a range of clusters of varying sizes. F: Contingency table of observed numbers of cluster ‘Neoblasts 2’ in small planarians. G: Contingency table of observed proportions of cluster ‘Neoblasts 2’ in small planarians. H: Contingency table of expected proportions of cluster ‘Neoblasts 2’ in small planarians. I. Resulting power analysis for the observed differences and effect size of Neoblasts 2 in small planarians. J: power analysis for all the abundance tests performed in the chi-square tests of Figure 4C. Dashed red line indicates power >0.8. K: power analysis for all the abundance tests performed in the chi-square tests of Figure 4B. Dashed red line indicates power >0.8. L: power analysis for all the abundance tests performed in the chi-square tests of Figure 4D. Dashed red line indicates power >0.8. M: scatter plot showing the relationship between cluster size and retrieved power of the tests in results from Figure 4C. Blue line represents regression line. Dashed red line indicates power >0.8.

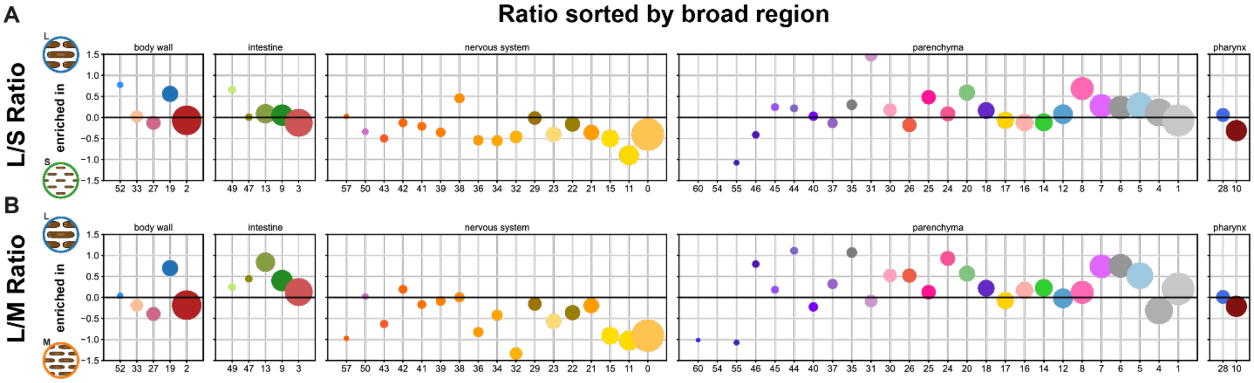

Fig. S6.

Variations in ratios of cell proportions by anatomical features. A: Percentage ratios of cell clusters in L vs S planarians sorted by body region. Dot size represents cluster size in cell number. B: Percentage ratios of cell clusters in L vs M planarians sorted by body region. In both plots cluster 54, corresponding to secretory 4 cells, lies outside of the plotted area due to a high enrichment in L, and cluster 60, corresponding to secretory 9 cells, lies outside of the plotted area due to a high enrichment in M.

.

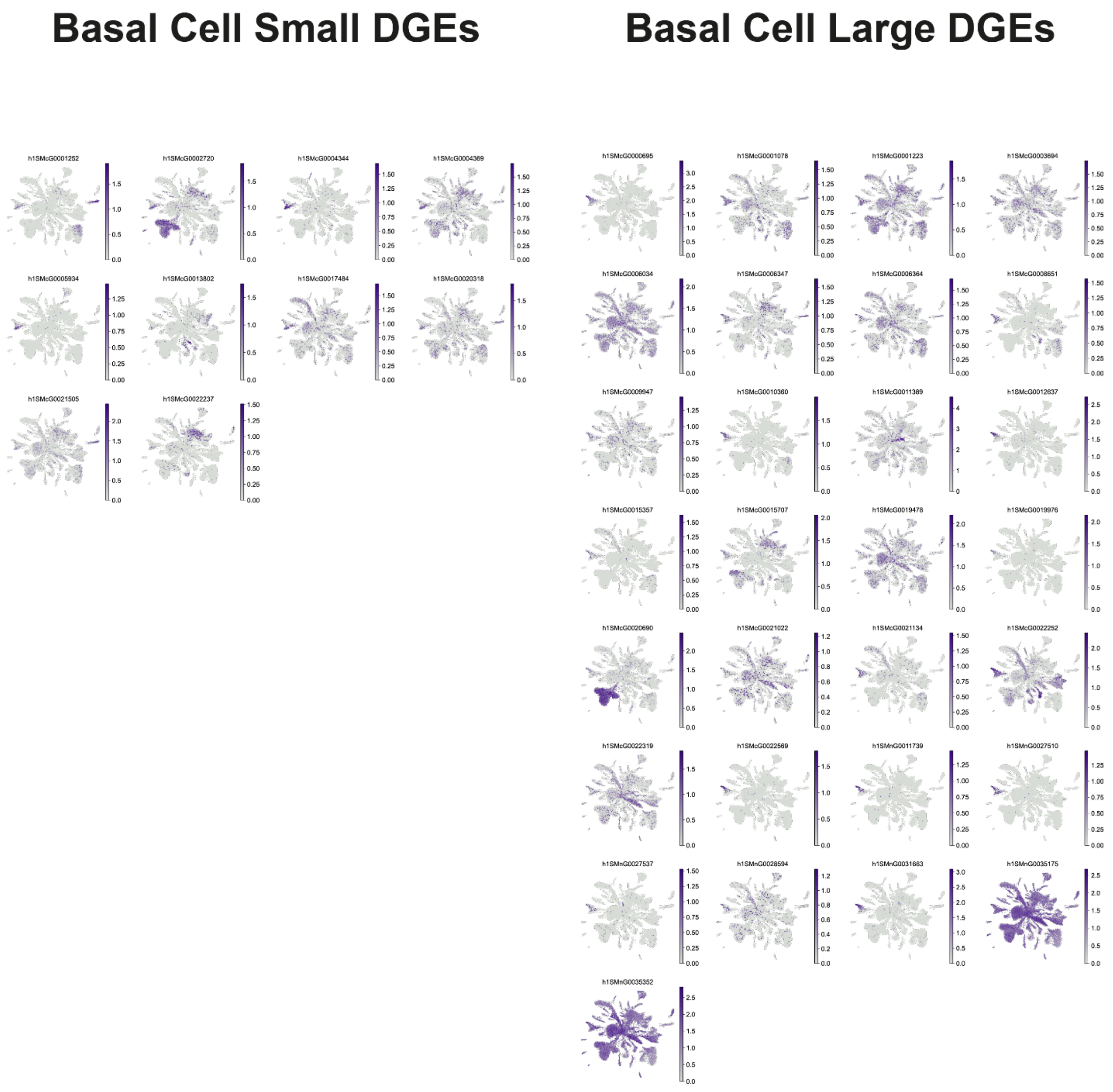

Fig. S7.

Differential gene expression analysis with DEseq2. Genes differentially regulated in S vs L planarians in basal cells.

.

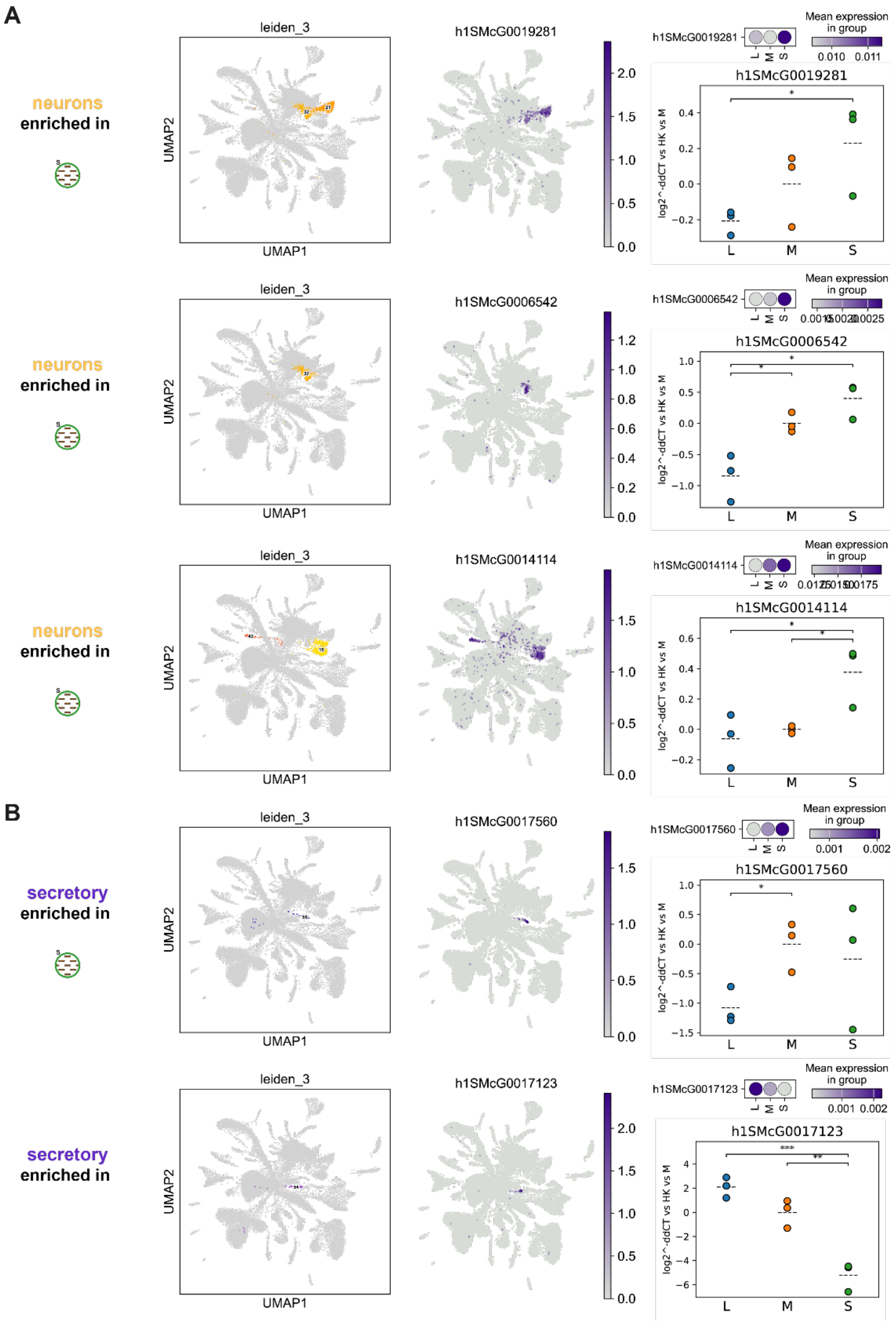

Fig. S8.

qPCR validation of the neuronal and secretory markers in L, M and S planarian RNA samples. A-B: UMAP projection of clusters (left), their markers (centre) and dotplot (right top) of the validated markers, and qPCR values (right bottom) of neuronal (A) and secretory (B) markers.

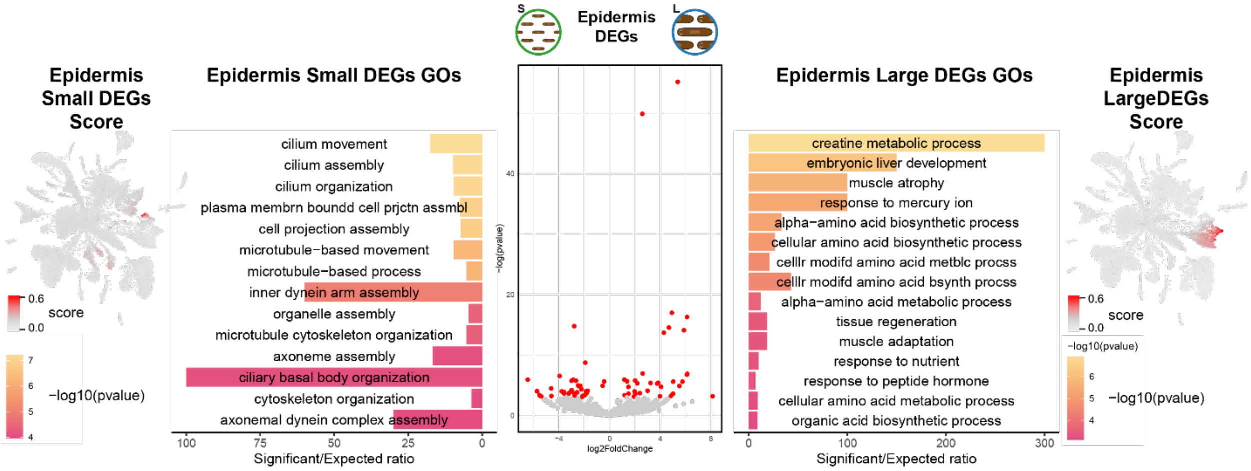

Fig. S9.

Differential gene expression in epidermis of L and S planarians. Differential gene expression analysis, GO term enrichment analysis and UMAP visualisation of gene scores, comparing the epidermis of L vs. S planarians. Bar size indicates ratio of significant/expected fraction of annotated genes with a given GO term. Colour gradient indicates adjusted p-value (Fisher's exact test). Scored expression of the group of differentially regulated genes in red.

Data S1.

Planarian L, M and S size measurements. Table of size measurements in mm^2 of animals selected, measured and single-cell sequenced in this experiment.

Data S2.

Leiden cluster annotation. Leiden clusters used in the data analysis, annotated with names, broad group membership, cell numbers per cluster, diagnostic markers, colours, and percentages.

Data S3.

***Schmidtea mediterranea* genome annotation**. *Schmidtea mediterranea* gene IDs and associated features.

Data S4.

Common cluster markers UMAP visualisations. UMAP plot visualisations of individual clusters and the markers that are common to Wilcoxon and Logistic Regression methods. Only the best 8 common markers are visualised. If the number of common markers is lower than 8, empty plots are presented.

Data S5.

Cluster Wilcoxon markers. Table of Wilcoxon markers, with gene annotations. If there are more than 50 significant markers, only the 50 with lowest p-values are presented.

Data S6.

Cluster Logistic Regression markers. Table of Logistic Regression markers, with gene annotations. Only the 50 with highest scores are presented.

Data S7.

Chi-squared statistical tests. Table of Chi-squared tests with p-values, residuals and significance levels.

Data S8.

qPCR primers. List of qPCR primers used in real-time qPCR validations.

Data S9.

DEseq2 Differential Gene Expression analyses. Table of Differential Gene Expression (DEseq2) analysis results, with for each broad cell type group.
